# Antimicrobial Agent Trimethoprim Influences Chemical Interactions in Cystic Fibrosis Pathogens via the *ham* Gene Cluster

**DOI:** 10.1021/acschembio.4c00562

**Published:** 2025-05-09

**Authors:** Jiangpeiyun Jin, Atharva S. Kulkarni, Andrew C. McAvoy, Neha Garg

**Affiliations:** † School of Chemistry and Biochemistry, 1372 Georgia Institute of Technology, 950 Atlantic Drive, Atlanta, Georgia 30332, United States; ‡ Center for Microbial Dynamics and Infection, Georgia Institute of Technology, 311 Ferst Drive, ES&T, Atlanta, Georgia 30332, United States

## Abstract

The fungus Aspergillus fumigatus and the bacterium Burkholderia cenocepacia cause fatal respiratory infections in immunocompromised humans and patients with lung disease, such as cystic fibrosis (CF). In dual infections, antagonistic interactions contribute to increased mortality. These interactions are further altered by the presence of antimicrobial and antifungal agents. However, studies performed to date on chemical interactions between clinical B. cenocepacia and A. fumigatus have focused on pathogens in isolation and do not include the most abundant chemical signal, i.e., clinically administered therapeutics, present in the lung. Here, we characterize small molecule-mediated interactions between B. cenocepacia and A. fumigatus and their shift in response to trimethoprim exposure by using metabolomics and mass spectrometry imaging. Using these methods, we report that the production of several small-molecule natural products of both the bacteria and the fungus is affected by cocultivation and exposure to trimethoprim. By systematic analysis of metabolomics data, we hypothesize that the B. cenocepacia-encoded *ham* gene cluster plays a role in the trimethoprim-mediated alteration of bacterial–fungal interactions. We support our findings by generating a genetically modified strain lacking the *ham* gene cluster and querying its interaction with A. fumigatus. Using comparative analyses of the extracts of wild-type and knockout strains, we report the inactivation of a bacterially produced antifungal compound, fragin, by A. fumigatus, which was verified by the addition of purified fragin to the A. fumigatus culture. Furthermore, we report that trimethoprim does not inhibit fungal growth, but affects the biochemical pathway for DHN-melanin biosynthesis, an important antifungal drug target, altering the pigmentation of the fungal conidia and is associated with modification of ergosterol to ergosteryl-3β-O-l-valine in coculture. This study demonstrates the impact of therapeutics on shaping microbial and fungal metabolomes, which influence interkingdom interactions and the expression of virulence factors. Our findings enhance the understanding of the complexity of chemical interactions between therapeutic compounds, bacteria, and fungi and may contribute to the development of selective treatments.

## Graphical abstract

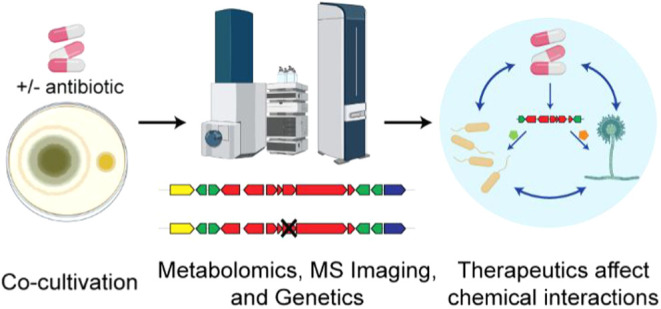

## Introduction

Cystic fibrosis (CF) is a progressive genetic disease caused by mutations in the cystic fibrosis transmembrane conductance regulator (CFTR) gene.^1^ During disease progression, the mucus in the CF patient’s lung becomes thick and sticky, facilitating opportunistic polymicrobial infections.^2^ Among bacterial infections, multidrug-resistant Staphylococcus aureus and Pseudomonas aeruginosa are the most prevalent; however Burkholderia cenocepacia complex (Bcc) species result in the most severe infection.^3,4^ Among Bcc, B. cenocepacia is one of the most prevalent species isolated from CF patients in the United States.^5^ Due to its inherent resistance to many commonly used antibiotics in the clinic and the ability of this genus for niche adaptation,^6,7^ B. cenocepacia infections are challenging to treat and the associated clinical outcomes are unpredictable and generally negative.^8^ During the course of recurrent infections, genetic diversification stems from and leads to a complex network of commensal, mutualistic, synergistic, competitive, or antagonistic interactions between the microbes^9,10^ and antibiotics,^11,12^ altering the community composition and antimicrobial susceptibility.^13^ Thus, the importance of studying microbes isolated from the lungs of CF patients in the presence of antibiotics is significant.

Our laboratory has previously characterized the response of B. cenocepacia to sublethal antibiotic concentrations present in the lung.^14–17^ The production of natural products, especially the antifungal compound fragin, was significantly upregulated in the presence of the antibiotic trimethoprim, which is used in the treatment of chronic *Burkholderia* infection in CF patients. These observations support the previous proposed roles of antibiotics as signaling molecules at sublethal concentrations affecting the microbial composition and structure in the natural and clinical environment.^12,18,19^ The induction of the antifungal compound fragin production by B. cenocepacia in the presence of trimethoprim led us to hypothesize that clinical antibiotics will influence chemical interactions between CF-associated *Burkholderia* and the fungal pathogens. The filamentous fungi, Aspergillus fumigatus, is the most common and prevalent fungal pathogen in CF^20,21^ and has been isolated from CF patients that are culture-positive for Bcc.^22^ Both Bcc bacteria and A. fumigatus are causes of concern among CF patients. They are known prolific producers of natural products^23,24^ and their interactions are likely affected by the presence of a sublethal concentration of drugs prescribed. However, no studies report on chemical interactions between *Burkholderia* and *Aspergillus* CF isolates in the presence of antibiotics, to the best of our knowledge. In light of our hypothesis, we believe that microbiological and chemical interactions between A. fumigatus and B. cenocepacia will be altered by antibiotics. Understanding the influence of antibiotics on interkingdom interactions may help in the development of potentiating treatments.

To decipher the chemical crosstalk between B. cenocepacia and A. fumigatus in the presence of the antibiotic trimethoprim, we applied ultrahigh-performance liquid chromatography tandem MS (UPLC-MS/MS)-based untargeted metabolomics coupled with matrix-assisted laser desorption ionization MS imaging (MALDI-ToF MSI).^25^ We describe bacterial and fungal natural products whose production was influenced by the exposure to trimethoprim and cocultivation. We report the inactivation of the bacterially produced antifungal compound fragin by A. fumigatus. Using growth inhibition assays, we observed that B. cenocepacia inhibits A. fumigatus only in the presence of trimethoprim. By generating a genetically modified strain of B. cenocepacia deficient in the biosynthesis of fragin, we report that the corresponding *ham* gene cluster plays a role in antibiotic-mediated inhibition of fungal growth and production of natural products by *Aspergillus*. In addition, we observed that the antibiotic trimethoprim, which does not inhibit A. fumigatus growth,^26^ alters pigmentation in fungal conidia, an important mediator of fungal virulence. These observations together highlight that advanced methods in chemical biology involving a combination of genetic, microbiological, and chemical techniques enable the discovery of clinically relevant phenotypes and underscore the importance of considering antibiotic exposures when studying polymicrobial and interkingdom interactions.

## Results and Discussion

### Metabolomic Profiling of Bacterial and Fungal Cells in Mono- and Cocultures

Trimethoprim is a clinically used antimicrobial agent against B. cepacia complex infection. To investigate metabolic interactions between B. cenocepacia K56–2 and A. fumigatus A1160+ (derived from the clinical isolate CEA10, Table S1) under clinically relevant conditions, the organisms were cocultured on an agar surface in the presence and absence of a sublethal concentration of trimethoprim (Figure 1). The appropriate monoculture and blank media controls were also included. The cultures were excised from agar and extracted with ethyl acetate (EtOAc), and the extracts were analyzed using the untargeted metabolomics workflow (Figure 1a,b). To visualize the spatial distribution of specialized metabolites, a section of the agar containing the colonies of both species was excised and subjected to MALDI-ToF MSI (Figure 1c).

**Figure 1:**
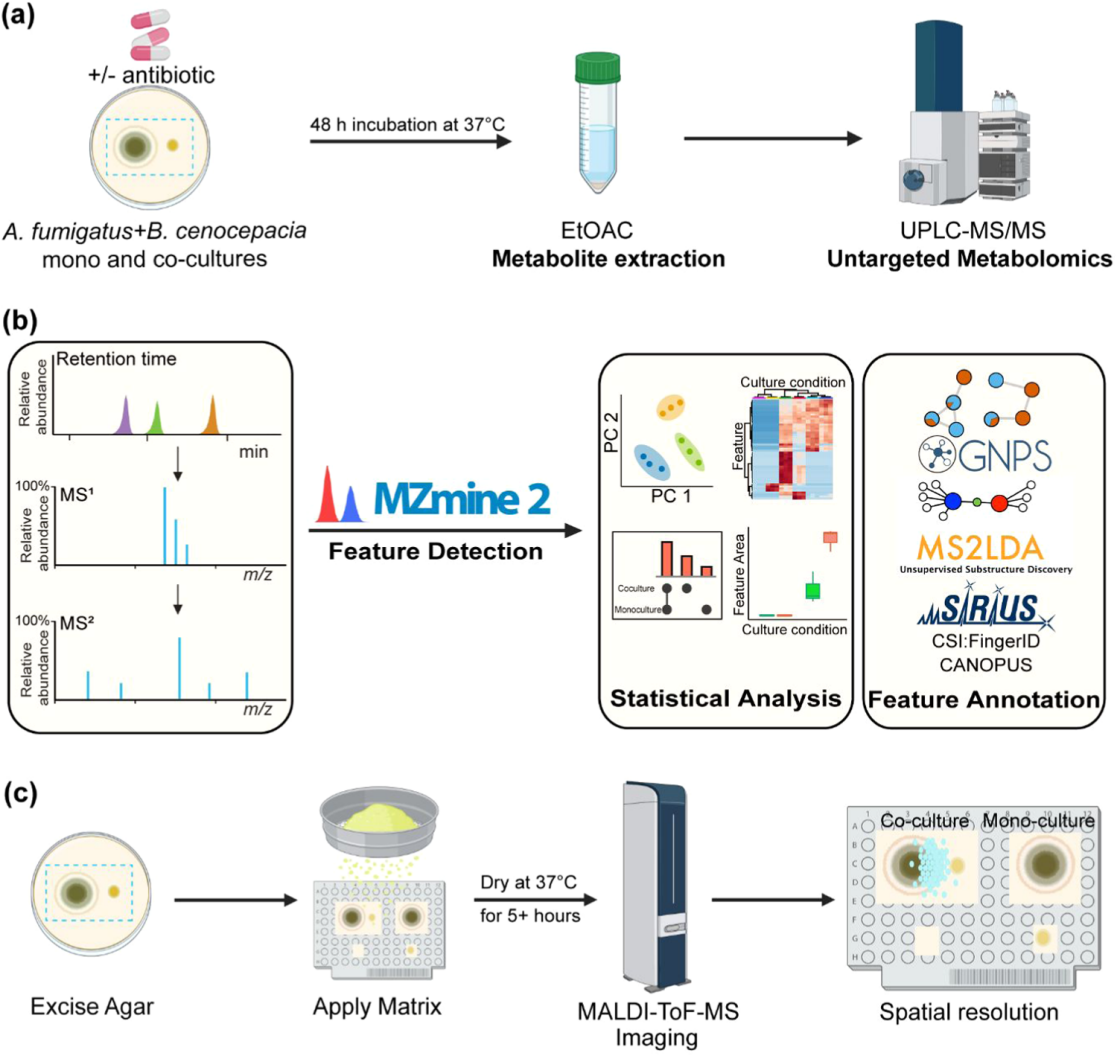
Overview of study design, sample processing, and metabolomics data acquisition and analyses. (a) Schematic representation of the workflow for culturing, extractions of samples, and UHPLC-MS/MS-based untargeted metabolomics data acquisition. (b) Data analysis workflow for untargeted metabolomics. (c) Schematic representation of the MALDI-ToF-MSI workflow. (Created with permission from BioRender.com).

Principal component analysis (PCA) and hierarchical clustering analysis (HCA) were performed to assess the shift in the detected metabolomes of B. cenocepacia and A. fumigatus cocultured in the presence and absence of sublethal trimethoprim. The metabolite features detected in culture media controls were removed prior to PCA. Thus, features such as the antibiotic trimethoprim supplemented to growth media do not contribute to the variance observed in PCA. Combined, the first and second principal components (PCs) captured 74% of the total variation (Figure 2a). The bacterial monoculture samples clustered separately from fungal monocultures and cocultures along PC1. Separation across PC2 correlated with the effect of exposure to trimethoprim, where the metabolomes of bacterial–fungal cocultures exposed to trimethoprim displayed the largest shift. Taken together, the PCA model reveals clear shifts in the metabolomic profiles of B. cenocepacia and A. fumigatus associated with both cocultivation and exposure to trimethoprim. Similarly, the HCA plot revealed the grouping of samples across two major clades (Figure 2b). The monoculture samples of B. cenocepacia clustered together in clade A, whereas all cocultures and fungal monocultures clustered in clade B. The samples corresponding to the coculture exposed to trimethoprim were further separated from the rest of the samples within clade B. The robust separation revealed by both PCA and HCA suggested that cocultivation affects the metabolomic profiles of both species and that the antibiotic exposure further influences the chemical crosstalk between B. cenocepacia and A. fumigatus.

**Figure 2:**
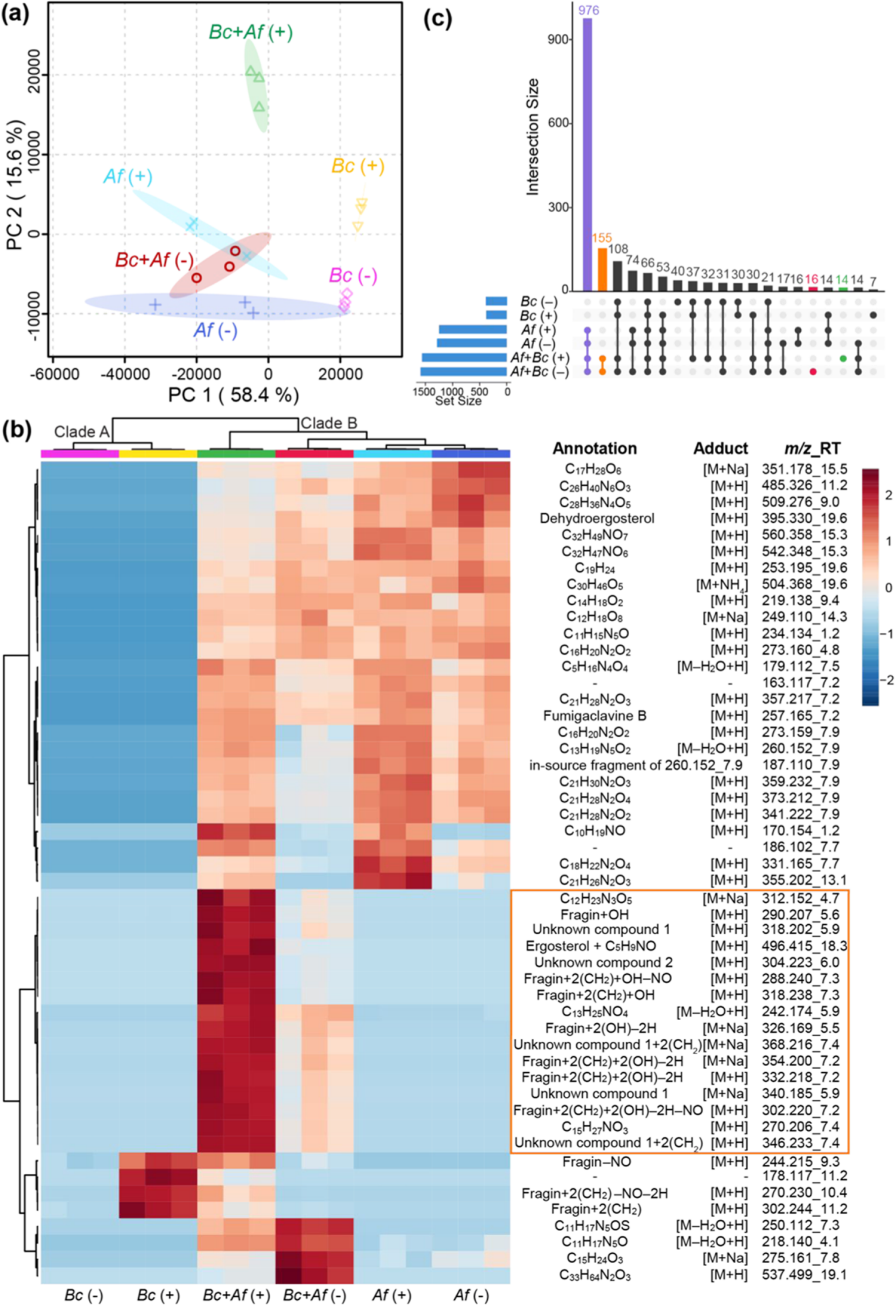
Metabolome profiling. (a) Principal component analysis of untargeted metabolomics data acquired on extracts of B. cenocepacia K56-2 (*Bc*) and A. fumigatus (*Af*) monocultures and B. *cenocepacia* K56-2 + A. fumigatus (*Bc* + *Af*) cocultures treated without (−) and with (+) trimethoprim. (b) Heatmap showing differences in relative abundances of the top 50 significant features identified via hierarchical clustering analysis. (c) UpSet plot analysis displaying the number of features detected across different growth conditions of antibiotic exposure and mono- and cocultures. For the sake of clarity, only the top 20 combinations of conditions in which the largest number of unique features were detected are shown.

To further survey the distribution of unique compounds detected across different culture conditions, an UpSet plot was generated (Figure 2c). Notably, 155 features corresponding to the second largest intersection were detected only in coculture conditions, regardless of the presence or absence of trimethoprim. An additional 14 metabolite features were exclusively detected in coculture samples in the presence of trimethoprim and 16 features in the absence of trimethoprim. Previous studies have reported that trimethoprim exposure induces secondary metabolite production in *Burkholderia*;^14,15,27,28^ this analysis further suggests that the interkingdom interactions between B. cenocepacia and A. fumigatus are affected by the exposure to clinical antibiotics. Such metabolic alterations raise the possibility that the resolution of the underlying infection caused by these pathogens may be influenced. Detailed analysis of specific metabolites contributing to the observed metabolomic shift is described below.

### Annotation of Significantly Differentially Produced Metabolites

B. cenocepacia and A. fumigatus produce a diversity of specialized metabolites, also called natural products.^14,23,29^ The metabolites detected exclusively in cocultures (UpSet plot, Figure 2b) as well as driving the observed clustering in HCA (Figure 2c) were prioritized for annotation. To annotate metabolite features driving the observed clustering in HCA, a heatmap of the top 50 significant metabolites was generated (Figures 2c and S1). To annotate these metabolites, we first generated a feature-based molecular network (FBMN) to connect metabolite features, represented as a node consisting of a retention time (RT) and a mass/charge ratio (*m*/*z*), based on structural similarity.^30^ We attempted annotations by searching both our in-house and publicly available mass spectral databases (Figure 3 and Table S2), providing insights into the chemical basis of interaction between the two species. In the absence of compound annotation, a chemical formula or chemical class predicted via CANOPUS^31^ is provided (Figure S2 and Table S3). Majority of the features uniquely detected in the coculture in the UpSet plot analysis belonged to natural product pathways based on the classification proposed by CANOPUS (Figure S2).

**Figure 3:**
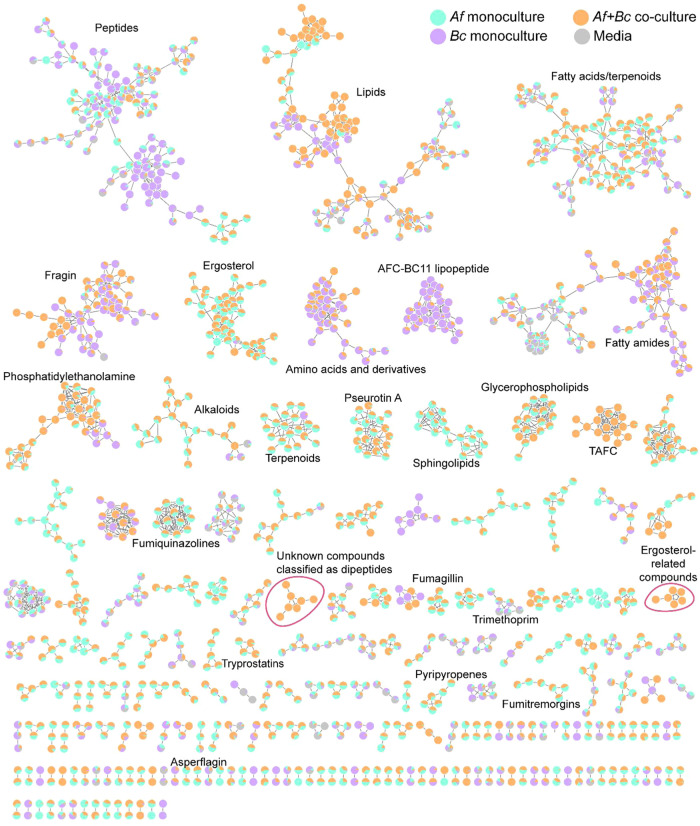
Feature-based molecular network of metabolomics data. UHLPC-MS/MS-based untargeted metabolomics data were processed and organized into 3539 nodes. By MS^2^ spectral matching with the GNPS library spectra, 210 nodes (6%) returned with putative annotations. Singletons were removed for the sake of clarity.

### Induction of the Fungal Siderophore in the Coculture

In the UpSet plot analysis, several features among the 155 features detected exclusively in the coculture exposed to trimethoprim either clustered together in FBMN or had a common substructure motif identified using MS2LDA^32^ (Figure 4a,b). One of the 155 features (*m/*z_RT 853.419_6.4) was annotated as triacetylfusarinin C (TAFC)^33^ using FBMN (Figures 4a, S3), which enabled the structural annotation of the common substructure motif 559 (Figure 4b). TAFC is an extracellular siderophore produced by A. fumigatus, facilitating the mobilization of environmental iron and is essential for its virulence in invasive aspergillosis models.^34^ Furthermore, we observed that the exposure to sublethal trimethoprim resulted in a significantly enhanced production of TAFC in the coculture in both iron-bound and unbound forms (Figure 4c,d). These observations were also supported in the MALDI MSI analysis. In the MALDI MSI data set, TAFC was detected exclusively in the coculture, predominantly at the periphery of the fungal colony and extending toward the bacterial colony (Figure 4d, top panel), with higher intensities observed in cocultures exposed to trimethoprim (Figure 4d, bottom panel). Other nodes in the cluster for TAFC (Figure 4a) include the intermediates *N*_2_-acetyl-*N*_5_-cis-anhydromevalonyl-*N*_5_-hydroxy-l-ornithine (*cis*-AAMHO, *m*/*z* 303.155, Figures 4a and S3) and dimeric *cis*-AAMHO (*m*/*z* 587.293, Figures 4a and S3) involved in the biosynthesis of TAFC. These intermediates showed identical detection patterns as TAFC. Iron is essential for the virulence of both B. cenocepacia and A. fumigatus,^35,36^ and initiation of infection is dependent on the pathogen’s ability to use host-complexed iron. In the case of *Aspergillus*, two high-affinity uptake systems including reductive (Fe^2+^) iron assimilation and siderophore-mediated iron acquisition are important. Among these, only siderophore-mediated acquisition was shown to be relevant for virulence in the invasive aspergillosis model.^37^ Consequently, efficient acquisition of iron is important for antagonistic interactions between the two pathogens. It has been reported that A. fumigatus adapts to iron limitation by upregulating siderophore biosynthesis.^38^ The production of TAFC was also reported to be induced during the interaction with P. aeruginosa, but the effect of antibiotics on TAFC production was not interrogated.^39^ Phenazines produced by *Pseudomonas* have an inhibitory effect against *Aspergillus*, and specifically, 1-hydroxyphenazine (1-HP) is the most active due to its ability to chelate iron, thereby causing an iron starvation response in A. fumigatus via the upregulation of TAFC production.^40^ The detection of TAFC exclusively under coculture conditions in our study suggests a competitive interaction between B. cenocepacia and A. fumigatus for iron. We hypothesized that a compound produced by B. cenocepacia likely affects the production of TAFC in cocultures and in response to exposure to trimethoprim. To explore this hypothesis, we performed systematic analyses of metabolomics data to query compounds produced by B. cenocepacia relevant to the induction of TAFC.

**Figure 4:**
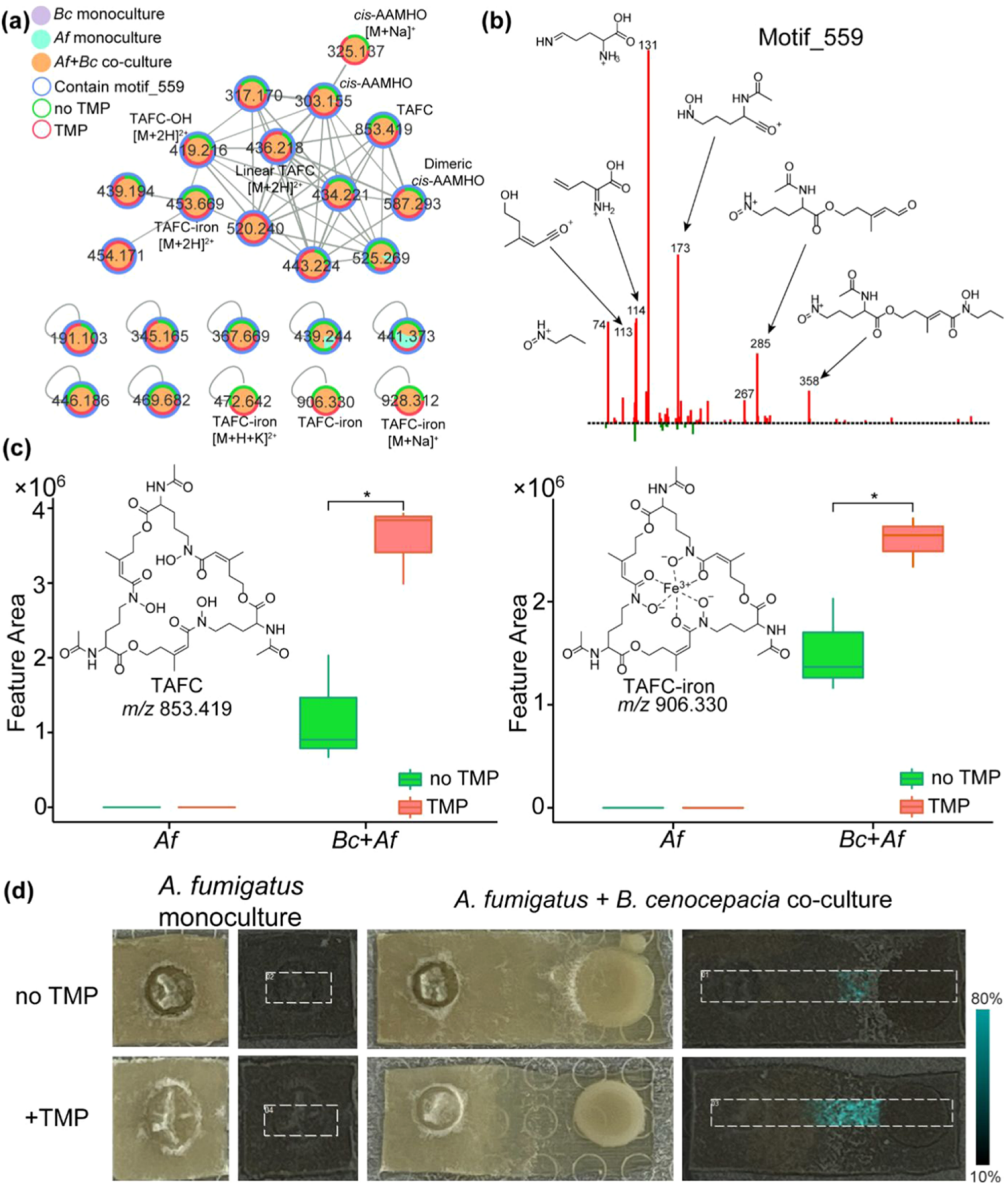
Fungal siderophore triacetylfusarinin C detected upon cocultivation of A. fumigatus and B. cenocepacia. (a) Molecular network of TAFC analogues connected via FBMN or MS2LDA. (b) Mass2Motif 559 corresponding to the TAFC substructure. (c) Boxplots of the relative abundance of TAFC and TAFC-iron complex in extracts derived from A. fumigatus monocultures and A. fumigatus + B. cenocepacia cocultures in the presence and absence of trimethoprim. Asterisks indicate the significant differences between the compared groups, as determined by the Kruskal–Wallis test with Dunn’s post-test (adjusted *p*-value <0.05). (d) MALDI-MSI-derived images for TAFC in mono- and cocultures. The visualization of the spatial distribution of TAFC is shown as a heatmap in false color (cyan, *m*/*z* 875.401, [M + Na]^+^). A white rectangle represents the measurement area used for data acquisition.

### Antifungal Compound, Fragin, and its Analogues in the Coculture

Among the top 50 features in HCA, a list of metabolite features was detected exclusively in coculture extracts and at a higher relative abundance under exposure to sublethal trimethoprim (Figure 2b, orange box). Many of these features clustered with the known antifungal compound fragin previously described from B. cenocepacia K56-2 (Figure S4).^14,41,42^ Although a significantly increased fragin production in the presence of trimethoprim is expected as reported in our previous studies,^14,15^ many of the analogues shown in the heatmap have not been described before. In this study, these analogues are detected only in the coculture and they predominantly represent hydroxylations and unsaturations in the acyl tail of fragin (Figures 2b and S5). The coculture and further exposure to antibiotics are likely to cause a shift in cellular fatty acid profiles, resulting in the production of unsaturated and hydroxylated fatty acids. The unsaturations and hydroxylations in fatty acids affect the membrane fluidity, which in turn can influence the functioning of membrane-bound receptors, ultimately altering signal transduction, ion transport, membrane potential, and receptor sensitivity.^43^ These fatty acids can also serve as substrates for the starter condensation domain of HamF that carries out the acylation in the biosynthesis of fragin resulting in the production of these analogues.^41,42^ Another possible explanation may be that these fragin analogues are now above the detection limit of the instrument due to the increased production observed under cocultivation.

In addition to fragin analogues, a feature with *m*/*z* 318.202 (unknown compound **1**) and its analogues were also captured in the heatmap and exclusively produced in cocultures. Furthermore, a significantly enhanced production was observed in the presence of trimethoprim (Figures 2b and S6). No annotations were obtained via our initial search of the spectral or compound library, literature, or *in silico* prediction, suggesting that these are likely previously unknown compounds produced by either *Aspergillus* or *Burkholderia* in cocultures. These compounds were classified as dipeptides based on the fragmentation pattern using CANOPUS. Trimethoprim was previously proposed to induce the production of extracellular proteases in B. thailandensis,^28^ which may be similarly responsible for the detection of these unique peptidic products in cocultures. However, the fragmentation pattern suggests that these might not be classical dipeptides generated from protein degradation but rather modified peptides. The annotations of these compounds are provided using Metabolite Annotation assisted by Substructure discovery and Stable Isotope Labeling by Amino acids in Cell culture (MAS-SILAC) analysis^16^ and are discussed below.

### Potential Modification of the Fungal Membrane Ergosterol

Both HCA and UpSet plot analyses revealed the detection of a cluster of unknown compounds exclusively in the extracts derived from trimethoprim-exposed cocultures (Figure S7a,b). Initial library searches within FBMN, manually in MS^2^-based mass spectral libraries and in compound databases, did not return any match to known metabolites. Thus, the MS2LDA substructure discovery-based approach was employed. The substructure motif, GNPS_motif_50, was present in multiple nodes in this unknown cluster, and it was connected to other FBMN clusters that were detected in the extracts of both A. fumigatus A1160+ monoculture and coculture samples. GNPS_motif_50 is a Mass2Motifs derived from the GNPS library, which has been annotated as a steroid core-related motif (Figure S7c). This annotation of the common substructural motif presented in multiple nodes, including some nodes in the unknown cluster, allowed the identification of the unknown cluster to be related to steroids (Figure S7c). Along with MS2LDA analysis, we employed the *in silico* tool CANOPUS to classify the unknown compounds into ClassyFire chemical classes.^31^ CANOPUS classifies them as steroids and steroid derivatives, providing additional confidence in annotating the unknown compounds as steroids. Among the clusters connected via GNPS_motif_50, one of the clusters was annotated as the ergosterol family of compounds. We confirmed the annotation of the feature with *m*/*z*_RT of 397.346_19.6 as the ergosterol by chromatographic and mass spectral comparison with the commercial analytical standard of ergosterol (*m*/*z*_RT 397.346_19.6, Figure S8a). The connection of the unknown cluster and ergosterol cluster through the shared Mass2Motif and comparison of the MS^2^ spectra suggests that the unknown cluster could potentially represent biotransformation of ergosterol in the coculture. These ergosterol-related features have a higher *m*/*z* than ergosterol, the *m*/*z* difference of 112.063 representing the addition of C_5_H_8_N_2_O to feature with *m*/*z* 509.410 or the addition of C_5_H_9_NO to feature with *m*/*z* 496.415. The chemical formulas based on HRMS are proposed to be C_33_H_53_NO_2_ and C_33_H_52_N_2_O_2_ for *m*/*z* 496.415 and 509.410, respectively. The alignment of the MS^2^ spectra of the unknown features with *m*/*z* 496.415 and 509.410 with the spectrum of ergosterol (*m*/*z* 397.346) further supports their structural relatedness to ergosterol (Figure S8b,c). MS^2^ comparison between ergosterol and the unknown features showed that the two fragment peaks at *m*/*z* 271.206 and 253.194 observed in ergosterol, resulting from the cleavage of the C–C bond between the side chain and the 5-membered ring and a consecutive dehydration from the *m*/*z* 271.206 fragment, were shifted by 2 Da in the unknown features (Figure S8d). This observation suggests that the modification is on the 3β-OH group of ergosterol.

Ergosterol is the most abundant sterol in the fungal cell membrane, which mediates membrane fluidity and permeability.^44^ Ergosterol is essential for fungal growth, survival, and virulence; therefore, the enzymes involved in the biosynthetic pathway of ergosterol have been used as targets for drug discovery.^45^ The uniquely detected ergosterol-related compounds can potentially be the biotransformation of ergosterol, which could be carried out by *Burkholderia* either in the coculture or by the fungus itself. Bioconjugation of natural products derived from two different partners in the coculture has been previously exemplified. For example, the conjugation between IQS (2-(2-hydroxyl-phenyl)-thiazole-4-carbaldehyde) produced by *Pseudomonas* and malleobactin B produced by *Burkholderia* led to the production of malleonitrone.^46^ To examine the possibility of conjugation between ergosterol and a metabolite from *Burkholderia* induced by trimethoprim, two experiments were performed. First, A. fumigatus was cultured next to the organic extracts of B. cenocepacia in the presence or absence of trimethoprim. Second, A. fumigatus was cultured in the growth medium supplemented with the spent medium of B. cenocepacia. However, ergosterol-related compounds were not detected under these experimental conditions. This observation supports the idea that a live bacterial cell is needed to induce the production of the unknown ergosterol-related compounds in the coculture. Additionally, it is worth noticing that the biosynthetic pathway of ergosterol is linked to that of TAFC, in which mevalonate is an important biosynthetic precursor for both the compounds.^47^ Previous studies report that the biosynthesis of ergosterol is downregulated, whereas TAFC production is upregulated during iron starvation, suggesting a significant interdependency between the biosynthesis of the two compounds.^47^ Since the unknown ergosterol-related compounds and induction of TAFC are observed under the same conditions, mevalonate limitation may alter the flux through these pathways. The putative annotations of ergosterol-related compounds are provided using MAS-SILAC analysis^16^ and are discussed below.

### Role of Trimethoprim and the *ham* Gene Cluster in Chemical Interactions between *Burkholderia* and *Aspergillus*

The metabolites synthesized by the *ham* gene cluster of B. cenocepacia K56-2, including fragin, and pyrazine *N*-oxides (PNOs) were induced in the presence of sublethal trimethoprim as previously described.^14,15^ In this study, the production of the aforementioned fungal compounds, TAFC, the potential ergosterol biotransformation product, and the unknown compounds classified as dipeptides showed the same trend when sublethal trimethoprim was added to the cocultures. While cocultivation of B. cenocepacia and A. fumigatus induced the production of the compounds, addition of trimethoprim further increased their production to a significantly higher relative abundance. The observation that compounds synthesized by the *ham* gene cluster are also induced by trimethoprim suggests that products of this cluster may play a direct or indirect role in the shifts observed in the production of fungal compounds in cocultures. The *ham* gene cluster comprises seven genes (*hamABCDEFG*) with the *hamC* gene encoding *p*-aminobenzoate *N*-oxygenase shown to be essential for the synthesis of fragin in B. cenocepacia.^41,42^ To investigate the potential role of the *ham* gene cluster in the bacterial–fungal chemical crosstalk, a markerless knockout in the *hamC* gene of B. cenocepacia K56-2 was generated and cocultured with A. fumigatus in the presence and absence of sublethal trimethoprim. Since we studied the effect of antibiotics in cocultures, a clean deletion was generated without incorporating any antibiotic resistance marker. Extracts derived from the coculture of A. fumigatus and B. cenocepacia K56-2 Δ*hamC* were subjected to UPLC-MS/MS data acquisition following the same untargeted metabolomics workflow described above. Cocultivation with B. cenocepacia K56-2 Δ*hamC* did not increase the production of TAFC in the presence of trimethoprim as compared to growth with the WT strain (Figure 5a). Furthermore, the compounds related to ergosterol and the unknown compounds classified as modified dipeptides were not detected in the extracts of A. fumigatus cocultivated with B. cenocepacia K56-2 Δ*hamC* (Figure 5b,c).

**Figure 5:**
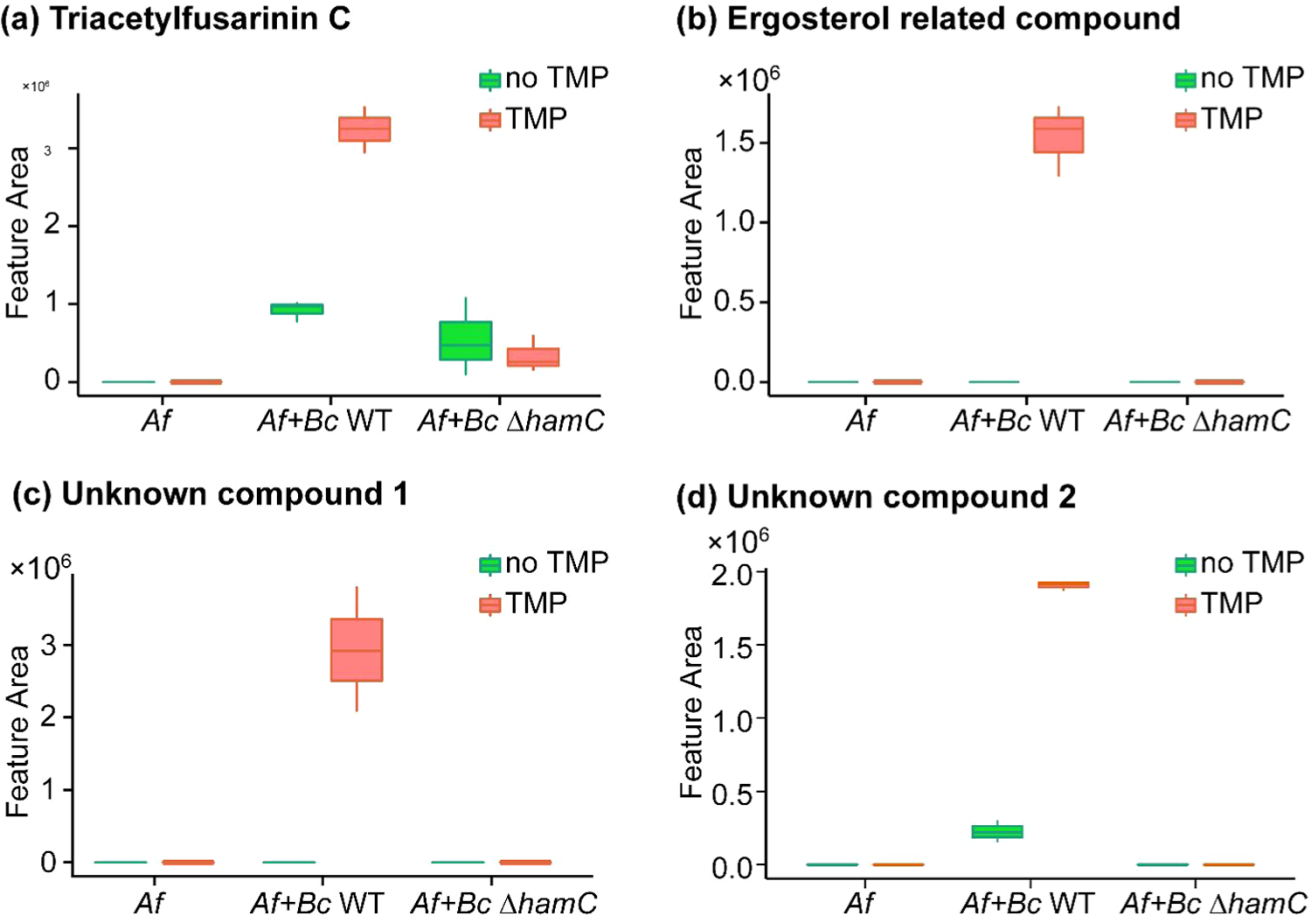
Shift in chemical interactions between B. cenocepacia and A. fumigatus in the absence of the functional *ham* gene cluster. Boxplots of the relative abundance of (a) triacetylfusarinin C, (b) the ergosterol-related compound with *m*/*z* 496.415, (c) the unknown compound **1** with *m*/*z* 318.202, and (d) the unknown compound **2** with *m*/*z* 304.223 in the extracts derived from A. fumigatus monocultures and cocultures with either B. cenocepacia WT or Δ*hamC* strains in the presence and absence of sublethal trimethoprim.

These observations imply that the molecules synthesized by the *ham* gene cluster play a role in influencing the production of the fungal siderophore TAFC, the unknown compounds classified as dipeptides, and the ergosterol-related compounds that were uniquely detected in A. fumigatus and B. cenocepacia cocultures. Exposure to sublethal trimethoprim leads to the induced expression of the *ham* gene cluster in B. cenocepacia, thereby altering the chemical interaction with A. fumigatus. A subset of the *ham* gene cluster was also identified to be homologous to the *Pseudomonas virulence factor* (*pvf*) cluster in *Pseudomonas entomophila* and the *mangotoxin-generating operon* (*mgo*) cluster in *Pseudomonas syringae*.^48–51^ The *pvf* and *mgo* gene clusters both encode the biosynthesis of natural products, including signaling molecules important for virulence.^49–57^ The diazeniumdiolate signaling molecule, valdiazen, is also detected in the cultures of a B. cenocepacia strain H111, but not in the strain K56-2 used in this study.^41,42,58^ In *Pseudomonas*, *pvf* and *mgo* regulate the production of several secreted proteins and natural products and affect the expressions of virulence-related genes, including siderophore transportation and the type VI secretion system.^51,52,56,57,59^ As homologues of *pvf* and *mgo* clusters, the *ham* gene cluster is likely to have the similar effect on *Burkholderia* virulence, implicating the potential to affect its interaction with the host and other microbes during infection. In a rat chronic respiratory infection model study, the *ham* gene clusters were shown to be dysregulated compared to a high-cell-density *in vitro* model, providing further insights into its importance for virulence, survival, and adaptation to the environment upon interaction with the host.^60^ Taken together, these prior studies and our findings suggest the importance of the *ham* gene cluster in shaping the physiology of *Burkholderia* during infection.

Our findings demonstrate the emerging function of antibiotics at sublethal concentrations as signaling molecules to stimulate gene expression in microorganisms and highlight the importance of studying the physiology of the pathogens under clinically relevant conditions. Leveraging untargeted metabolomics allowed us to describe the current biological reality of these microorganisms by considering the impact of therapeutic compounds to which pathogens are exposed during infection.

### Stable Isotope Labeling-Guided Annotation of Features Classified as Dipeptides

A neutral loss of 76.027 Da was conserved in the MS^2^ spectra of all of the unknown compounds classified as dipeptides within the molecular network cluster (Figure 3, circled pink, and Figure S9). The chemical formula of this neutral loss is predicted to be CH_4_N_2_O_2_ (Figure S9). We proposed that this neutral loss likely contains a diazenium group based on the chemical formula and mass defect. Given that the diazeniumdiolate group is present in fragin, we hypothesized that these compounds may be related to fragin. To test this hypothesis, we applied MAS-SILAC. Since valine is the precursor for biosynthetic products of the *ham* gene cluster, we predicted that the substructure corresponding to the neutral loss of 76.027 Da is derived from valine. Thus, we cultured A. fumigatus and B. cenocepacia in M9 minimal media supplemented with 0.4% glucose as a carbon source and isotope-labeled valine (^13^C_5_,^15^N,D_2_-valine).

The features with *m/*z 318.202 (compound **1**) and *m*/*z* 304.223 (compound **2**) both exhibit an increase in mass of 7.021 Da (Figure S9a,b). These mass shifts in the unknown compounds are 1 Da less than the incorporation of the entire ^13^C_5_,^15^N,D_2_-valine, and this difference of 1 Da could potentially be due to proton exchange during the biosynthesis process. Notably, the neutral loss of 76.027 Da shifted to 77.025 Da, corresponding to the loss of a ^15^N from valine (Figure S9a,b). The stable isotope labeling experiment demonstrates that these unknown compounds are synthesized from valine. This observation along with the chemical formula for the neutral loss (CH_4_N_2_O_2_) and the fact that HamC, which is essential for the biosynthesis of the diazeniumdiolate group,^58^ is required for the detection of these compounds suggests that the neutral loss corresponds to a methylated diazeniumdiolate group. To extract all of the features containing the methylated diazeniumdiolate group displaying a neutral loss of 76.027 Da and all of the features containing a diazeniumdiolate group displaying a neutral loss of 29.998 Da, we employed MassQL and generated a molecular network on the output (Figure 6a). The output of this analysis allowed us to identify a fragin analogue that is methylated at the *O* ^2^ position of the diazeniumdiolate group (Figure 6a, *O* ^2^-Me-fragin and its sodium adduct, green nodes and Figure 6b). Since the *O* ^1^-methylation of diazeniumdiolate has been characterized to be unstable, and *O* ^1^-methylation could still generate the NO loss (NL of 29.998 Da) through the cleavage of the N–N bond,^61^ we proposed that the methylation is specifically on the *O* ^2^ position of the diazeniumdiolate group of fragin. The *O* ^2^-methylation can stabilize the diazeniumdiolate and prevent the release of NO.^62^ Thus, a neutral loss of 76.027 Da is observed instead of 29.998 Da (NO loss) in the MS^2^ of the compounds that contain a methyl group at the *O* ^2^ position (Figure 6b). Based on this, we annotated the feature with *m*/*z* 288.228 as *O* ^2^-Me-fragin and related features to be analogues of *O* ^2^-Me-fragin. By propagating network annotation, we annotated several analogues of *O* ^2^-Me-fragin (Figure 6a). *O* ^2^-Me-fragin has been previously synthesized by Gademann group and was shown to be inactive in the antifungal assay against the fungus *Fusarium solani*.^42^ Thus, the observed *O* ^2^-Me-fragin under cocultivation implies a defensive response by A. fumigatus through inactivation of the diazeniumdiolate functional group, preventing copper binding by the compound and the disruption of the antifungal activity. To the best of our knowledge, this is the first reported *O* ^2^-methylation of diazeniumdiolate through a biosynthetic process.

**Figure 6:**
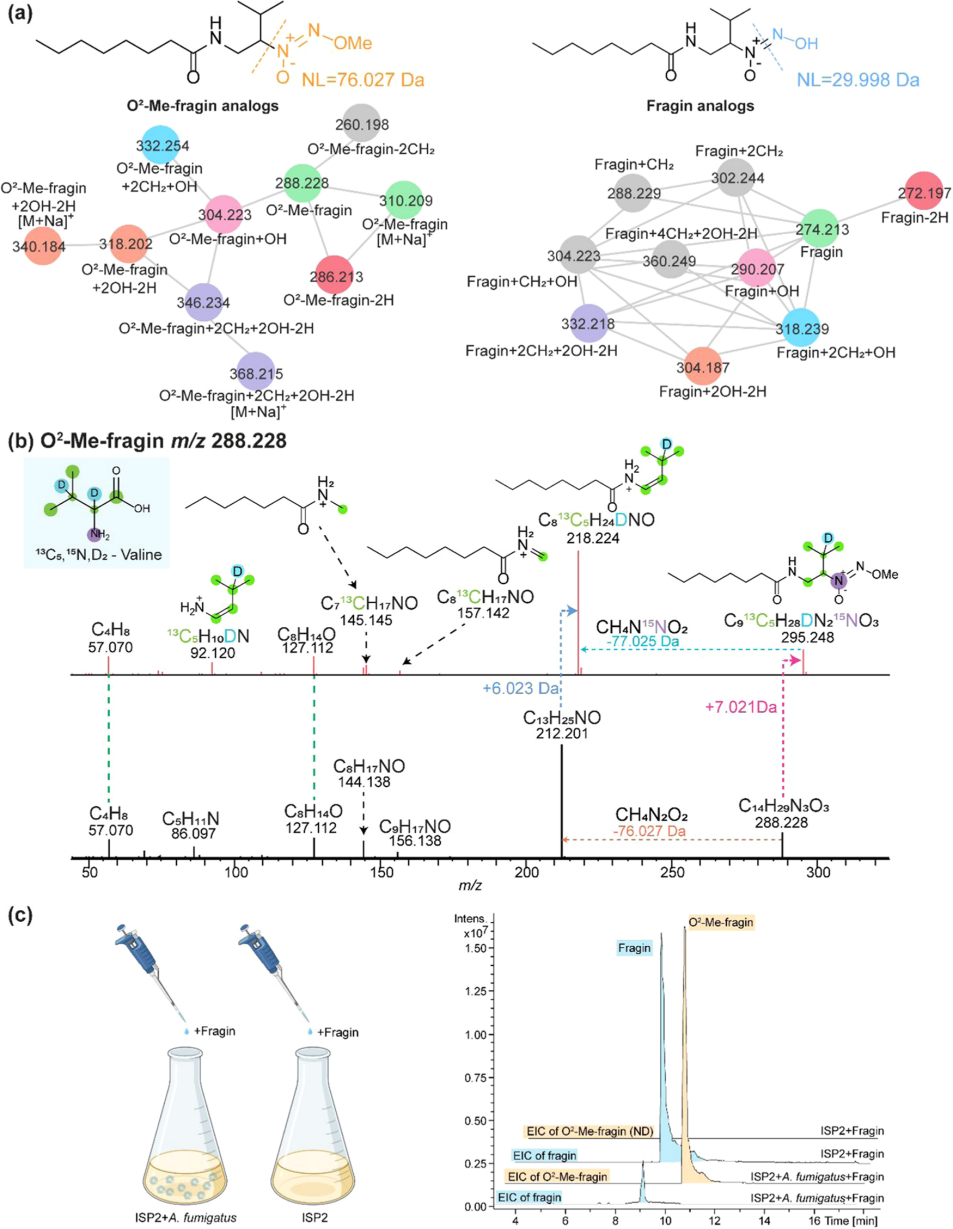
MAS-SILAC-guided annotation of fragin-related compounds. (a) Molecular network of features extracted using query language MassQL (see Methods), containing NL values of 76.027 Da (left) and 29.998 Da (right). Nodes with the same color represent fragin analogues with the same acyl chain. (b) Annotated MS^2^ spectrum of unlabeled *O* ^2^-Me-fragin and labeled *O* ^2^-Me-fragin (using the incorporation of ^13^C_5_,^15^N,D_2_-valine). (c) EICs of fragin and *O* ^2^-Me-fragin detected in culture extracts of A. fumigatus supplemented with purified fragin and extracts of a media control without A. fumigatus, but supplemented with purified fragin. ND refers to not detected.

To support our proposition that A. fumigatus inactivates fragin, we purified fragin from B. cenocepacia (Figure S10) and supplemented the A. fumigatus culture with purified fragin. Almost a complete conversion of fragin to *O* ^2^-Me-fragin was observed in the presence of A. fumigatus (Figure 6c, EIC trace of *O* ^2^-Me-fragin in yellow), whereas no conversion of fragin was observed in the absence of A. fumigatus cells. Future investigations using activity-guided protein fractionation and proteomics will aid in the identification of methyl transferase involved in this biotransformation.

### Stable Isotope Labeling-Guided Annotation of Ergosterol-Related Compounds

The ergosterol-related compounds exhibited an increase of 8.026 Da when cultured in M9 minimal media containing ^13^C_5_,^15^N,D_2_-valine (Figure 7). The shifts in the MS^2^ spectra of ergosterol-related compounds were consistent with the incorporation of ^13^C_5_,^15^N,D_2_-valine into their structures. The two distinct MS^2^ peaks at *m*/*z* 196.170 and *m*/*z* 142.123 also exhibited the same mass shift of 8.026 Da, indicating that these two fragments contain ^13^C_5_,^15^N,D_2_-valine (Figure 7a). These observations suggest that the ergosterol-related compounds resulted from the conjugation of valine and ergosterol. Additionally, the *m*/*z* of the other MS^2^ peaks corresponding to the steroid core did not shift in the isotope-labeled compounds, which further suggests that the side chain of ergosterol is modified. All of the carbons of ergosterol and related compounds were labeled when ^13^C_6_-glucose was supplemented as the carbon source in the culture media (Figure S11). Based on this information, we proposed that the feature with *m*/*z* 496.415 is likely ergosteryl-3β-O-l-valine (Figure 7). Indeed, many bacteria remodel their membranes by aminoacylating lipids enzymatically.^63^ Similarly, fungi including A. fumigatus have been shown to aminoacylate ergosterol with glycine^64^ and aspartate^65^ at the 3β-OH position, resulting in the formation of ergosteryl-3β-O-l-glycine and ergosteryl-3β-O-l-aspartate. Aminoacylated lipids and sterols are proposed to play an important role in host–bacteria, polymicrobial, and microbe-drug interactions.^63^ Thus, our observations of aminoacylation of ergosterol in the presence of antibiotics during bacterial-fungal cocultures shed light on the complexity of interkingdom chemical interactions. Future work using biochemical methods, such as protein fractionation of bacteria and fungi, will identify the mechanism involved in ergosterol modification.

**Figure 7:**
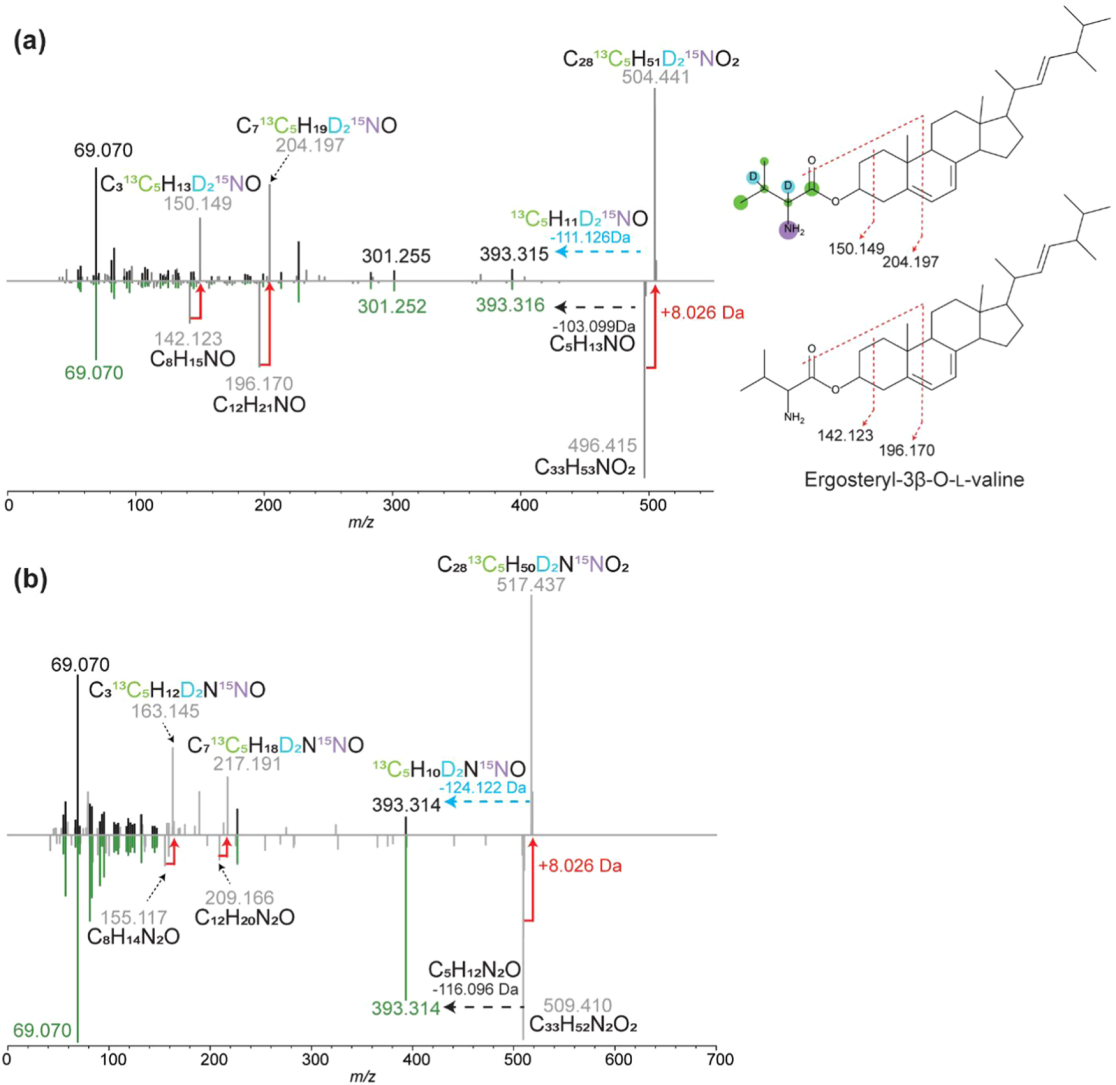
MS^2^ spectral analysis of labeled and unlabeled ergosterol-related compounds and incorporation of ^13^C_5_,^15^N,D_2_-valine. (a) Annotated MS^2^ spectrum of unlabeled and labeled ergosterol-related compounds with *m*/*z* 496.415 and (b) annotated MS^2^ spectrum of unlabeled and labeled ergosterol-related compounds with *m*/*z* 509.410.

### Inhibition of A. fumigatus Growth by B. cenocepacia

Antimicrobials at sublethal concentrations, present during infection, have been acknowledged to act as intermicrobial signaling molecules modulating gene expression and host response.^12,66^ Many studies have endeavored to characterize the effects of sublethal antibiotics on gene expression related to virulence,^67^ motility,^68^ biofilm formation,^69^ and stress response^70^ in microorganisms. Since fragin, a diazeniumdiolate metallophore with antifungal activity,^41,42^ is produced in the presence of trimethoprim, we tested the effect of sublethal trimethoprim on the antifungal activity of B. cenocepacia. In the agar diffusion growth inhibition assay, we observed significant inhibition of A. fumigatus growth by B. cenocepacia only when the assay was supplemented with trimethoprim (Figure 8a). Furthermore, increasing the trimethoprim concentration resulted in stronger inhibition of A. fumigatus growth by B. cenocepacia, evidenced by the widening gap between fungal and bacterial colonies. However, no inhibition of A. fumigatus A1160+ growth was observed in the coculture with the B. cenocepacia Δ*hamC* strain (Figure 8b). Similar to our observations, the B. cenocepacia K56-2 strain was previously shown to have no inhibition against A. fumigatus in the synthetic sputum medium.^71^ Here, we demonstrated that the inhibitory activity against A. fumigatus is influenced by the antibiotic trimethoprim and is linked to the production of fragin by B. cenocepacia. This antifungal phenotype in the presence of trimethoprim indicates that the competitive interkingdom interactions between bacterial and fungal CF pathogens are altered by clinical antibiotics, emphasizing the significance of studying such interactions in the presence of therapeutic compounds.

**Figure 8:**
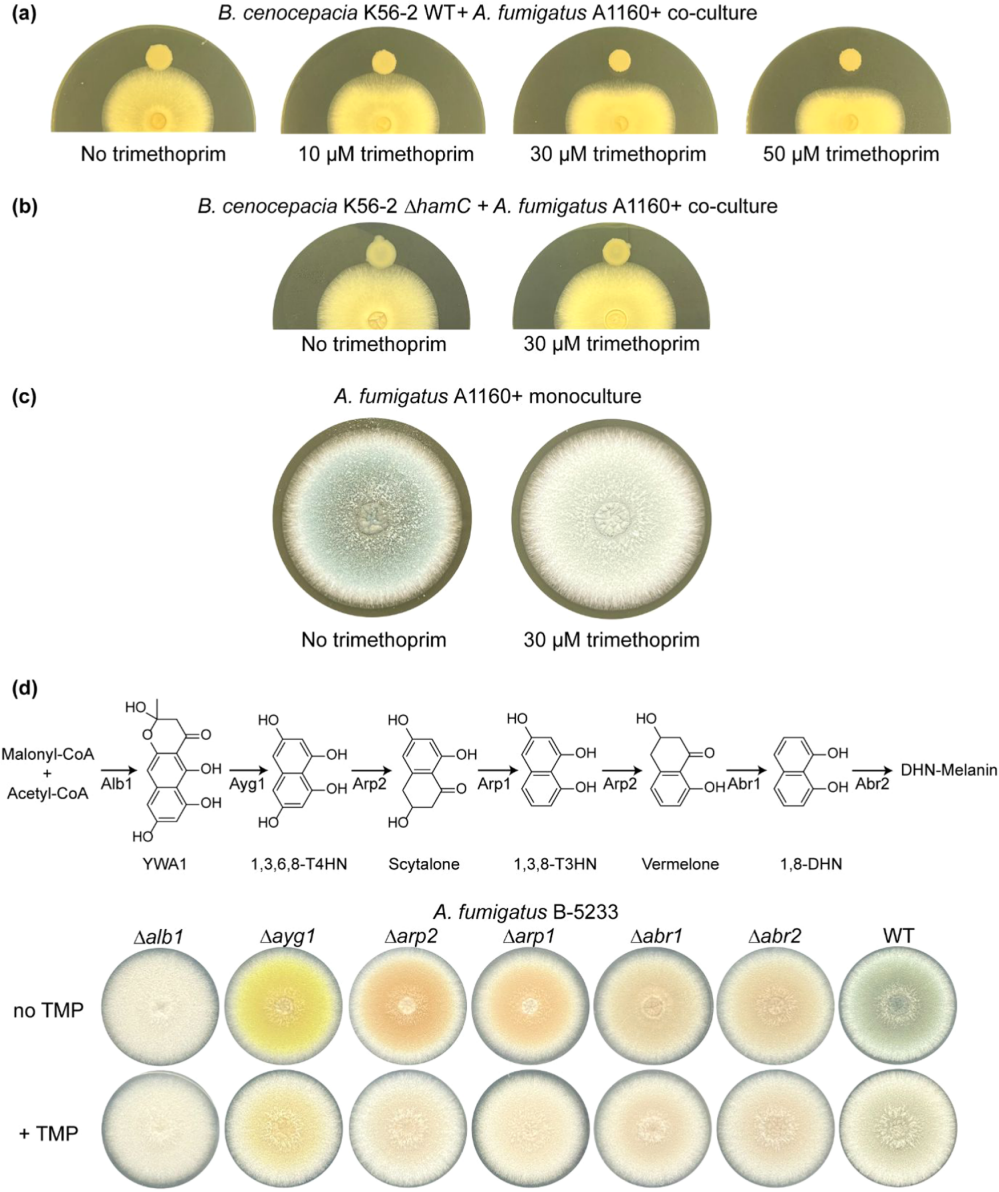
Phenotypic changes of A. fumigatus upon exposure to trimethoprim at a sublethal concentration. (a) Assessment of the antifungal activity of B. cenocepacia against A. fumigatus in the absence and presence of sublethal trimethoprim. Supplementing the culture media with trimethoprim at sublethal concentrations led to a significantly increased inhibition of A. fumigatus by B. cenocepacia. (b) Loss of antifungal activity of B. cenocepacia lacking the *hamC* gene against A. fumigatus A1160+. (c) Dysregulation of fungal pigment production in the presence of trimethoprim. Images of A. fumigatus A1160+ in the monoculture on solid agar supplemented with and without trimethoprim are shown. (d) Dysregulation of the fungal DHN-melanin biosynthesis pathway in the presence of trimethoprim. Biosynthetic pathway of DHN (dihydroxynaphthalene)-melanin is shown in the top panel. Images of the A. fumigatus B-5233 WT strain and six single-gene deletions (Δ*alb1*, Δ*ayg1*, Δ*arp2*, Δ*arp1*, Δ*abr1*, Δ*abr2*) cultured on solid agar supplemented with (bottom row) and without (top row) trimethoprim are shown. T4HN: tetrahydroxynaphthalene and T3HN: triydroxynaphthalene.

### Dysregulated Production of DHN-melanin in A. fumigatus by Trimethoprim

Serendipitously, we observed a difference in the pigmentation of A. fumigatus A1160+ conidia under exposure to sublethal trimethoprim (Figure 8c). The A. fumigatus colonies without trimethoprim had a blue-green color with powdery conidiophores, but the fungal colony appeared to be significantly lighter in color in the presence of trimethoprim (Figure 8c). The PCA plot also displayed a shift in the metabolome of A. fumigatus in trimethoprim-exposed monocultures (Figure 2b). A. fumigatus produces a wide range of toxic secondary metabolites to humans and animals, known as mycotoxins, providing survival benefits for them to adapt to particular environments.^72,73^ To explore whether the production of additional fungal compounds is affected by the presence of trimethoprim, we attempted annotations of natural products produced by A. fumigatus. Using various strategies for compound annotation, we annotated several known fungal mycotoxins in our data set (Figures 3, S12–13 and Table S4). For instance, identification of the fungal mycotoxins was facilitated by analysis of the genome using the fungal version of antiSMASH (Table S5),^74^ querying natural product databases using CEU Mass Mediator^75^ and matching the experimental MS^2^ spectrum and spectra available in the GNPS library^76^ or literature. The production of these mycotoxins did not show significant differences in the presence of trimethoprim, with the exception of YWA1 (Figure S14). YWA1 is a naphthopyrone,^77^ and it is the first intermediate product in the biosynthetic pathway of DHN-melanin.^78^ The naphthopyrone class of compounds possesses a wide variety of biological activities including antibacterial,^79^ immunomodulatory,^80^ antiproliferative,^81^ and cytotoxic properties.^82^ The blue-green pigment is attributed to the production of 1,8-dihydroxynaphthalene melanin (DHN-melanin) in the conidia cell wall of A. fumigatus.^83^ Melanin has been known to be crucial for the survival and longevity of fungal propagules.^84^ DHN-melanin is reported to play a role in immune evasion and is crucial in the control of A. fumigatus infections in both humans and mice.^83^ Therefore, DHN-melanin is important for the virulence of A. fumigatus and the biosynthetic pathway is considered an important drug target.^83,85^ The biosynthesis of DHN-melanin in A. fumigatus employs six genes in BGC (Figure 8d, top panel), with mutations in each of the genes resulting in different pigmentations of the conidia color.^86^ Notably, mutation in the *alb1/pksP* gene involved in the biosynthesis of YWA1 displayed a white conidia colony. Similarly, deletion of the genes encoding the transcription factors that regulates *pksP* expression resulted in a lighter green colony but not as white as the Δ*pksP* mutant strain.^87^ The appearance of the lighter or whiter colony of A. fumigatus on agar supplemented with sublethal trimethoprim suggests that the biosynthesis of DHN-melanin is likely affected by trimethoprim. To further investigate the effect of trimethoprim exposure on DHN-melanin production, we cultured A. fumigatus B-5233 WT and six single-gene deletion strains (Δ*alb1*, Δ*ayg1*, Δ*arp2*, Δ*arp1*, Δ*abr1*, Δ*abr2)* ^86^ in the absence and presence of trimethoprim. Notably, WT and all six single-gene deletion strains of A. fumigatus B-5233 appeared to be lighter in color when cultured in the presence of trimethoprim (Figure 8d). It has been previously shown that trimethoprim does not inhibit the growth of *Aspergillus* species.^26^ In agreement, the colony diameter and sporulation ability of all A. fumigatus strains were not affected by the presence of sublethal trimethoprim. Our observations suggest that it can impact the fungal morphology and physiology without affecting growth. Nevertheless, reduced production of DHN-melanin could potentially diminish the virulence of A. fumigatus, affect its interactions with immune cells, and weaken the competitive advantage when interacting with B. cenocepacia. Further mechanistic investigation of this phenotypic change is important to understand the effect of antimicrobial compounds on conidia formation in A. fumigatus.

In summary, the complexity of chemical and biological milieu within the lungs of CF patients affects the interaction networks between the organisms present and plays a crucial role in shaping the phenotypes of the associated pathogens. The therapeutics taken during treatment represent a large component of this milieu and are distributed heterogeneously within the tissue creating hotspots of infection. A shift in chemical interactions affected by the sublethal antimicrobial compounds is seldom studied in dual infection models. Advancements in chemistry-first approaches, including untargeted metabolomics, are being increasingly employed to close this knowledge gap. Here, by applying untargeted metabolomics approaches, we demonstrated that the production of natural products by CF-associated bacterial and fungal pathogens is altered by antibiotic exposure during cocultivation. MALDI-ToF MSI facilitated the direct visualization of the spatial distribution of metabolites involved in the bacterial-fungal crosstalk. These observations enabled the generation of a hypothesis to identify the underlying pathways mediating these interactions. We validated the hypothesis by generating scarless gene deletion mutants and analysis of modified and wild-type interactions using metabolomics. This multipronged strategy allowed us to identify a gene cluster, namely, the *ham* gene cluster in Burkholderia cenocepacia, the expression of which plays an important role in bacterial-fungal interactions and in the production of virulence factors by the cocultivated fungus. We highlight that chemistry-first approaches such as metabolomics pave the way for the potential identification of novel enzymes via activity-guided protein fractionation in future studies. Lastly, unexpectedly, we observed a reduction in pigmentation of the fungus during growth in the presence of antibacterial trimethoprim. Systematic analysis of fungal metabolomes in the presence of trimethoprim allowed us to identify the impact of trimethoprim on the production of the natural product YWA1 and the corresponding pathway for fungal DHN-melanin biosynthesis, which plays a role in this phenotype. Thus, our findings highlight that the presence of antibiotics in the CF lung environment is a significant factor influencing the virulence factor production of not only bacteria but also fungi, thereby modulating chemical interactions between the microorganisms. Such interspecies interactions contribute to the complexity and accuracy in the prediction of clinical outcomes and promote disease progression in CF patients. Our findings underscore the importance of investigating polymicrobial interactions under conditions that more accurately reflect the CF lung environment, which will be essential for characterizing pathogenic phenotypes and advancing effective treatments for microbial infections.

## Materials and Methods

### Strains and Materials

The strain B. cenocepacia K56-2 belongs to the highly transmissible and epidemic ET12 lineage and was isolated from a CF patient.^88^ A. fumigatus A1160+ used in this study is a derivative of the clinical isolate CEA10 ^89,90^ and A. fumigatus B-5233 was isolated from a patient with fatal invasive aspergillosis.^86^ Strains, plasmids, and primers used in this study are listed in Supplementary Table S1. All oligonucleotides were purchased from Integrated DNA Technologies. The MALDI matrix, trimethoprim, and sulfadimethoxine were obtained from Sigma-Aldrich. Hexakis­(1H,1H,2H-perfluoroethoxy) phosphazene was purchased from SynQuest Laboratories. All other media components for ISP2 were sourced from BD chemicals.

### Microbial Growth Conditions

For bacterial culturing, frozen glycerol stocks of each strain were aseptically streaked on LB agar plates and incubated at 37 °C overnight. Once individual colonies were visible, a single colony was used to inoculate 5 mL of LB broth. These cultures were incubated overnight at 37 °C while being shaken at 200 rpm and used as a starter culture to inoculate three biological replicates of ISP2 agar supplemented with or without 30 μM trimethoprim. Trimethoprim (12 mM stock) was prepared fresh in dimethyl sulfoxide (DMSO).

Overnight cultures of B. cenocepacia K56-2 wild-type and the Δ*hamC* mutant strain lacking the gene for the HamC protein were pelleted by centrifuging at 4700*g* for 3 min and decanting to separate the supernatant from the cell pellet. Bacterial pellets were washed with fresh LB broth twice and adjusted to an OD_600_ value of 1.0. For untargeted metabolomics samples, 5 μL of B. cenocepacia K56-2 WT or Δ*hamC* strain suspension was inoculated 1.5 cm away from a 5 μL inoculum of A. fumigatus on ISP2 agar. Similarly, B. cenocepacia and A. fumigatus were inoculated separately as monocultures. Samples were incubated for 48 h at 37 °C. For antifungal activity assay, two 10 μL of each bacterial suspension were spotted 2.5 cm apart on ISP2 plates (standard Petri plates 100 × 15 mm) and were allowed to dry for 15 min before incubating overnight at 37 °C. The next day, 10 μL of the A. fumigatus spore stock was inoculated at the center of ISP2 plates with the spotted bacteria. The plates were incubated at 37 °C and photographed after 48 h of growth. Culturing in M9 minimal media was performed using the same protocol.

### Construction of the B. cenocepacia K56–2 Δ*hamC* Knockout

An in-frame markerless deletion for knocking out the *hamC* gene was generated in B. cenocepacia K56-2 using the allelic exchange method.^91^ Briefly, primer pairs P1/P2 and P3/P4 (Supplementary Table S1) were used to amplify regions flanking *hamC*, assembled into the suicide vector pMo130-Tc using a Gibson assembly, and transformed into E. coli DH5α. The transformants were selected on LB agar plates containing 10 μg/mL tetracycline. A mist of 0.45 M pyrocatechol that gives positive transformants a yellow-colored appearance was used to screen bacterial colonies. The plasmid was isolated from the positive transformants, verified by sequencing, and transformed into the donor strain E. coli SM10 by electroporation. The donor E. coli SM10 strain carrying the plasmid was used for conjugation with the recipient K56-2 using puddle mating.^92^ For this, early log-phase cultures of the donor and recipient were combined in a 3:1 ratio, and 100 μL of this mixture was incubated on LB agar plates at 30 °C overnight. The cells were resuspended in phosphate-buffered saline (PBS) and merodiploids were selected by plating on Vogel-Bonner minimal medium plates supplemented with 100 μg/mL of tetracycline and containing citrate as the sole carbon source to select against E. coli. The plates were then incubated at 37 °C for 2 days and then sprayed with pyrocatechol mist to confirm the integration of the plasmid into the genome of the recipient. PCR-verified merodiploids were chosen for counterselection by streaking merodiploids on no-salt LB (NSLB) agar plates supplemented with 20% sucrose followed by incubation at 37 °C for 3 days to force a second recombination event yielding an unmarked *hamC* deletion in K56–2. To eliminate colonies that were still merodiploids, pyrocatechol was sprayed, and colonies that turned yellow were excluded from further screening. Successful deletion was confirmed using PCR with primer pairs P5 and P6 and sequencing using the primer pair P7/P8. A mist of 0.45 M pyrocatechol that gives positive transformants a yellow appearance was used to screen bacterial colonies wherever appropriate.

### Sample Preparation for Metabolomics

Metabolites were extracted from cultures with ethyl acetate (EtOAc). A predefined rectangular region containing either the monoculture or both species was excised from the agar (Figure 1). The agar was chopped into small pieces using a scalpel and transferred to a 50 mL Falcon tube. The extractions were performed by adding 10 mL of EtOAc to each tube containing the chopped agar and vortexed for 30 s. The resulting extracts were centrifuged at 3000 × *g* for 3 min and the organic solvent was transferred into a scintillation vial with a glass pipet. Two serial extractions were performed with 10 mL of EtOAc and pooled. The EtOAc extracts were concentrated *in vacuo* and stored at −20 °C until further analysis.

### Ultrahigh-Performance Liquid Chromatography Tandem Mass Spectrometry (UHPLC-MS/MS) Data Acquisition

Dried extracts were dissolved in 300 μL of 80% MeOH (LC-MS grade) containing 1 μM sulfadimethoxine as an internal standard. A 20 μL sample volume was analyzed using a 1290 Infinity II UHPLC system (Agilent Technologies) coupled to an ImpactII ultrahigh-resolution Qq-ToF mass spectrometer (Bruker Daltonics) equipped with an ESI source. Chromatographic separation was achieved on a Kinetex 1.7 μm C18 reversed-phase HPLC column (50 × 2.1 mm). Solvent A consisted of water with 0.1% (v/v) formic acid, and solvent B consisted of MeCN with 0.1% (v/v) formic acid. The gradient used for chromatographic separation consisted of 5% B and 95% A for 2 min, a linear increase to 95% B over 17 min, a hold for 3 min, a linear decrease to 5% B in 1 min, and a hold for 1 min with a constant flow rate of 0.5 mL/min throughout. For MS spectra acquisition, the instrument was set to capture features from *m*/*z* 50–2000 Da in positive ion mode. An external calibration with the ESI-L Low Concentration Tuning Mix (Agilent Technologies) was performed prior to data collection, and hexakis­(1H,1H,2H-perfluoroethoxy) phosphazene was used as an internal lock-mass calibrant throughout the run. Ion source parameters were set to 4500 V for the capillary voltage, 2 bar for the nebulizer gas pressure (N_2_), 200 °C for the ion source temperature, 9 L/min for the dry gas flow, and a spectral rate of Hz for MS^1^ and 6 Hz for MS^2^. For MS^2^ data acquisition, the eight most intense precursor ions per MS^1^ scan were selected for MS^2^ fragmentation. A basic stepping function was used to fragment ions at 50 and 125% of the CID calculated for each *m*/*z*, with a timing of 50% for each step. A basic stepping of collision RF of 550 and 800 Vpp with a timing of 50% for each step and transfer time stepping of 60 and 150 μs with a timing of 50% for each step was employed. The MS/MS active exclusion parameter was set to 2 and the active exclusion was released after 30 s. The mass of the internal lock-mass calibrant was excluded from the MS^2^ acquisition. A wash method was run to clean the column after every six samples, followed by a solvent blank to monitor for any residual carryover. A mixture of six compounds (amitriptyline, sulfamethazine, sulfamethizole, sulfachloropyridazine, sulfadimethoxine, and coumarin-314) was run after every six samples to check the system performance.

### Data Processing, Feature-Based Molecular Networking, and Feature Annotation

The acquired raw LC-MS/MS spectral data were converted to the mzXML format in Bruker Compass Data Analysis software and preprocessed with MZmine2 ^93^ (v.2.5.3) for feature detection. Data files were batch-processed and filtered using parameters described previously.^15^ A quantification table with ion intensities for each feature across each sample was exported (.csv format) for statistical analyses, and a mgf file was generated for batch analysis with SIRIUS (v.5.6.2). Additionally, a feature quantification table and the corresponding list of MS^2^ spectra linked to MS^1^ features was exported using the “Export for GNPS-FBMN” module and the two files were submitted to the Global Natural Products Molecular Networking (GNPS) platform along with a metadata file (.txt format) containing sample information, and a feature-based molecular network was created. Molecular networks and parameters used are accessible at the following links: https://gnps.ucsd.edu/ProteoSAFe/status.jsp?task=3bb1c7ce1779456d981c0161a29f08b4. Networks were exported to Cytoscape for visualization.^94^ Next, MS2LDA analysis was implemented to enable further annotation of analogues of the same compound that were not present as connected nodes in the network by searching the recurring patterns of common MS^2^ fragments and neutral losses, which correspond to molecular substructures.^32^ MS2LDA analysis and the parameters used are accessible at the following links: https://gnps.ucsd.edu/ProteoSAFe/status.jsp?task=23df229f393b42a9b70ef487c5df8e7f. Boxplots were created for features of interest in RStudio using the nonparametric Kruskal–Wallis test with Dunn’s post-test for significance levels between groups. Adjusted *p*-values (the Benjamin-Hochberg method) of 0.05 or less were considered statistically significant. The UpSet plot was generated with the UpSet module on Intervene Shiny App^95^ and used to identify unique features detected in conditions of interest.

Annotations of compounds were performed using a suite of data analysis tools. Spectral matching was employed through GNPS′ online server; compound class and structural predictions were completed *in silico* with SIRIUS^96^ (v.5.6.2) equipped with CANOPUS^31^ and CSI:FingerID.^97^ SIRIUS was implemented to predict molecular formulas and develop fragmentation trees to aid with the manual annotation of MS^2^ spectra using the default settings for a qToF instrument. CSI:FingerID was applied to predict structural fingerprints of unknown features, which were then queried against all available chemical databases to provide a ranked list of possible known structures. CANOPUS was deployed to classify features into ClassyFire^98^ compound classes, providing biological insight into detected compounds eluding structural annotation. A literature search for known compounds produced by *Burkholderia* spp. and other closely related bacteria was conducted to aid with annotating features of interest not identified via GNPS spectral library searching. Thus, the annotations are supported through analysis of biosynthetic gene clusters, MS^2^ spectral comparison with previously published spectra, spectra available in public libraries, comparison with spectral data acquired on analytical standards in this study, and manual annotation of MS^2^ fragments resulting in level 2 compound annotations.^99^

MassQL is a user-friendly structured query language-inspired tool that allows scientists lacking the expertise in coding to search large data sets. In this study, MassQL was applied to identify all metabolite features with the neutral loss of 29.998 and 76.027 Da, using the query input as “QUERY scaninfo­(MS2DATA) WHERE MS2NL = (29.998 OR 76.0273):TOLERANCEMZ = 0.01”.

### Fermentation, Extraction, and Compound Purification

B. cenocepacia K56-2 was inoculated at an OD_600_ of 0.05 in 1 L flasks containing 100 mL of LB containing 30 μM trimethoprim. Cultures were incubated at 37 °C with shaking at 200 rpm for 24 h. Two serial extractions of the culture (2 L) were performed with 2 L of EtOAc each, and the extracts were pooled, dried, and fractionated using C18 Seppak resin (Phenomenex, 10*g*). The dried extract was resuspended in water and the column was eluted with 10 *C–V* of each 20% MeCN, 40% MeCN, and 100% MeCN. The 40% fraction contained fragin, as assessed by HPLC-MS. This fraction was dried *in vacuo*, resuspended in 2:3 MeOH/H_2_O, and further purified by semipreparative HPLC using a 5 μm Phenomenex Luna C18 column (250 × 10 mm^2^) operating at a flow rate of 2 mL/min with a gradient of 5–100% MeCN (in H_2_O) over 25 min. Elution was monitored with a UV detector monitoring the run at 190 and 238 nm.

One-dimensional (1D) and two-dimensional (2D) NMR spectra were recorded on a Bruker Avance III HD 500 MHz NMR in CDCl_3_ and calibrated using the residual undeuterated solvent as an internal reference. The NMR data for fragin has been deposited to NP-MRD^100^ (ID: NP0350930).

### MALDI-ToF MS Imaging

An inoculum (5 μL each) of B. cenocepacia K56-2 WT and A. fumigatus was spotted 1.5 cm apart on ISP2 agar (1 mm thick, 10 mL of agar poured in a standard 100 mm × 15 mm Petri dish) and incubated at 37 °C for 48 h. Both B. cenocepacia K56-2 WT and A. fumigatus cultures were spotted by themselves as monocultures. The agar containing the cells and the uninoculated agar were then excised from the Petri dish and gently placed on a Bruker Daltonics ground steel MALDI 96 anchor target plate as described previously.^25^ A cotton swab dampened with LC-MS-grade acetonitrile was used to gently remove the hyphae of A. fumigatus before applying the matrix. The recrystallized and finely powdered matrix of 1:1 2,5-dihydroxybenzoic acid:α-cyano-4-hydroxycinnaimic acid was applied to the sample using the sieve method and dried for a minimum of 5 h at 37 °C following the general procedures described by Yang et al.^101^ For calibration of the instrument, a peptide calibration standard (Bruker Daltonics Inc., 8206195) was mixed with the matrix and spotted on the target plate next to the dried agar slice. All samples were imaged using rapifleX MALDI-ToF at a scan range of 25 × 25 μm resulting in a field size of 104 × 104 μm in reflectron positive mode in the mass range of 200 to 3520 *m*/*z*. The laser intensity and detector gain were optimized for individual experiments, and spectra were acquired using a M5 defocus as the laser setting. Data were analyzed by using Bruker Daltonics FlexImaging 5.0.

## Supplementary Material



## Notes

**Supplementary figures:** Heatmap showing top 100 features; chemical diversity of unique metabolite features detected; MS^2^ mirror plots of triacetylfusarinin C and MS^2^ spectra of its biosynthetic intermediates; feature-based molecular network of the hydroxylated fragin analogues; MS^2^ spectra comparison of hydroxylated fragin analogues; molecular network and fragmentation pattern of compounds **1** and **2**; MS^2^ mirror plots of ergosterol; MS^2^ mirror plots of mycotoxins matched with the spectra in the GNPS library; MS^2^ mirror plots of mycotoxins matched with the spectra in literature; and extracted ion chromatogram of YWA1. **Supplementary tables**: Bacterial and fungal strains, plasmids, and primers used in the study; putative annotation of metabolite features showed in the heatmap; list of metabolite features detected exclusively in extracts derived from cocultures; list of known fungal mycotoxins detected in this study; biosynthetic gene clusters present in B. cenocepacia and A. fumigatus strains analyzed in this study; and supplementary references (PDF)
