# Supplementary material for: Antimicrobial Agent Trimethoprim Influences Chemical Interactions in Cystic Fibrosis Pathogens via the ham Gene Cluster
Source: ACS Chem Biol. 2025 May 9;20(6):1153–70. doi: 10.1021/acschembio.4c00562 (PMC12186260; doi:10.1021/acschembio.4c00562)
Supplement: Supplementary file 1 [file cb4c00562_si_001.pdf]

Antimicrobial agent trimethoprim influences chemical interactions in cystic fibrosis pathogens via *ham* gene cluster

Jiangpeiyun Jin[a], *Atharva Kulkarni*[a], *Andrew C. McAvoy*[a], *Neha Garg*[a,b,*]

[a]School of Chemistry and Biochemistry, Georgia Institute of Technology, 950 Atlantic Drive, Atlanta, Georgia 30332, United States

[b]Center for Microbial Dynamics and Infection, Georgia Institute of Technology, 311 Ferst Drive, ES&T, Atlanta, GA 30332, United States

KEYWORDS. Cystic Fibrosis, *Burkholderia, Aspergillus,* untargeted metabolomics, antibiotic, natural products, signaling, conidia

Table of Contents

Figure S1. Heatmap showing top 100 features driving the separation revealed in HCA 3

Figure S2. Chemical diversity of unique metabolite features detected in extracts of coculture samples......4

Figure S3. MS^2 mirror plots of triacetylfusarinin C and MS^2 spectra of its biosynthetic intermediates........5

Figure S4. Feature-based molecular network of the hydroxylated fragin analogs 6

Figure S5. MS^2 spectra comparison of hydroxylated fragin analogs 7

Figure S6. Molecular network and fragmentation pattern of the unknown compounds **1** and **2** 8

Figure S7. Analysis of ergosterol related compounds 9

Figure S8. MS^2 mirror plots of ergosterol and the related compounds 10

Figure S9. MAS-SILAC guided annotation of the fragin related features 11

Figure S10. Structural analysis of purified fragin 12

Figure S11. Analysis of $^{13}C_6$-glucose labeled ergosterol and related compounds. 13

Figure S12. MS^2 mirror plots of mycotoxins matched with spectra in GNPS library 14

Figure S13. MS^2 mirror plots of mycotoxins matched with spectra in literature.......................................15

Figure S14. Extracted ion chromatogram of YWA1 (*m/z* 277.071) 15

Table S1. Bacterial and fungal strains, plasmids, and primers used in the study 16

Table S2. Putative annotation of metabolite features showed in heatmap 17–18

Table S3. List of metabolite features detected exclusively in extracts derived from co-cultures..........19–23

Table S4. List of known fungal mycotoxins detected in this study 24

Table S5. Biosynthetic gene clusters present in *B. cenocepacia* and *A. fumigatus* strains analyzed in this study. 24–27

Supplementary References...28

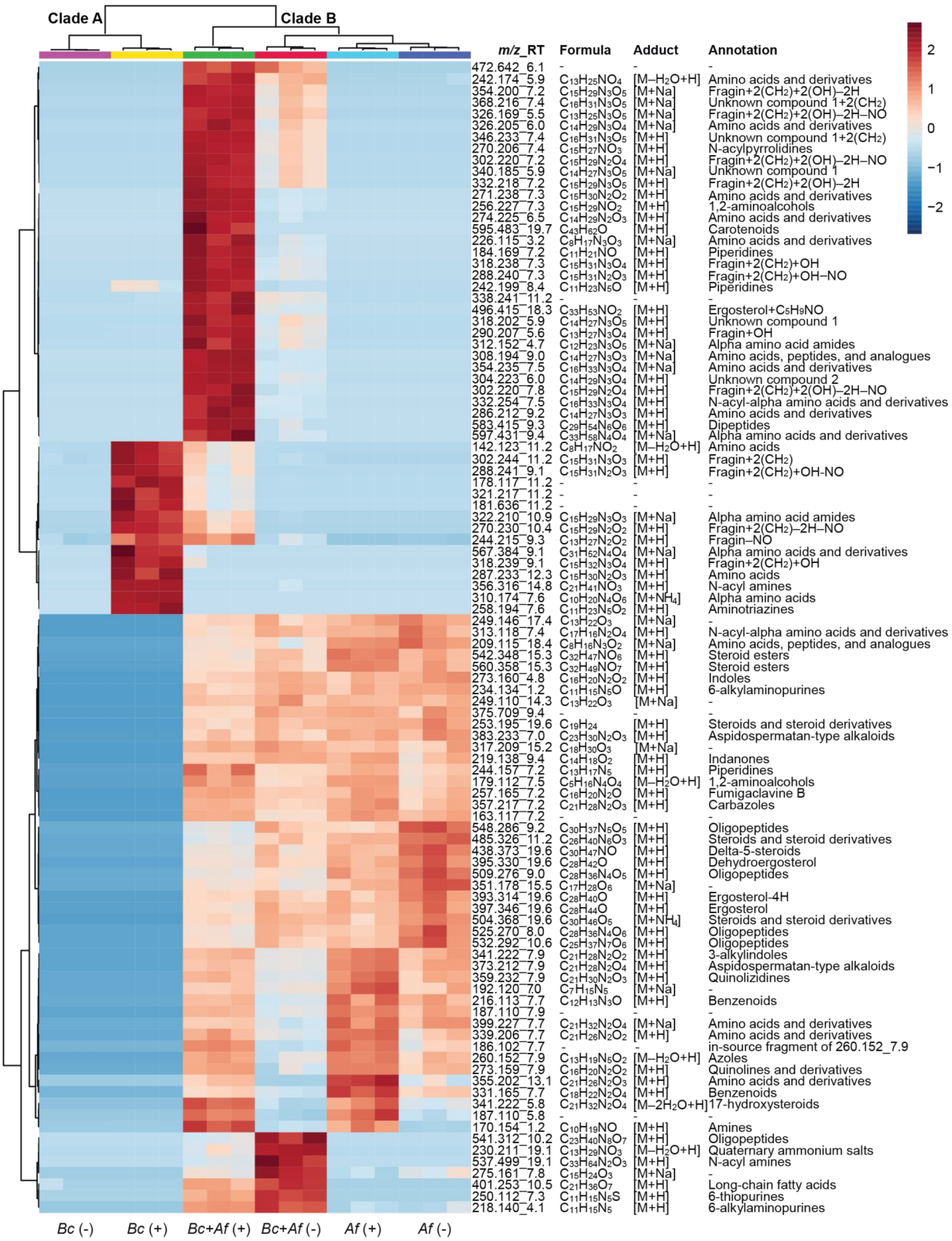

Supplementary Figure S1. Heatmap of the top 100 features for hierarchical clustering analysis.

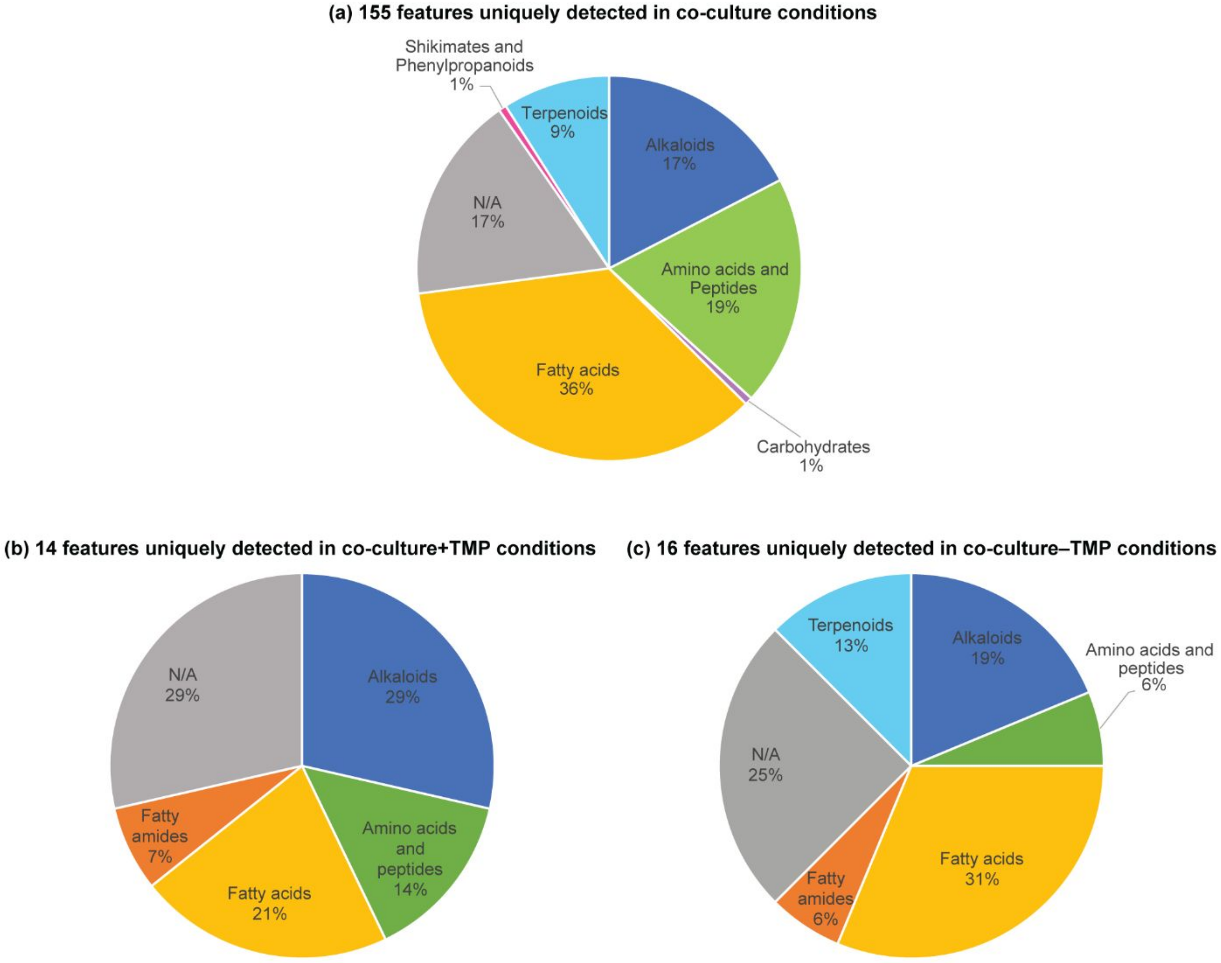

Supplementary Figure S2. Chemical diversity of metabolite features detected uniquely in extracts derived from co-culture samples revealed in UpSet plot. Natural product pathways of these metabolite features were determined using CANOPUS.[1] (a) 155 features detected in extracts derived from co-culture samples. (b) 14 features detected in extracts derived from co-culture with trimethoprim conditions. (c) 16 features detected in extracts derived from co-culture without trimethoprim samples.

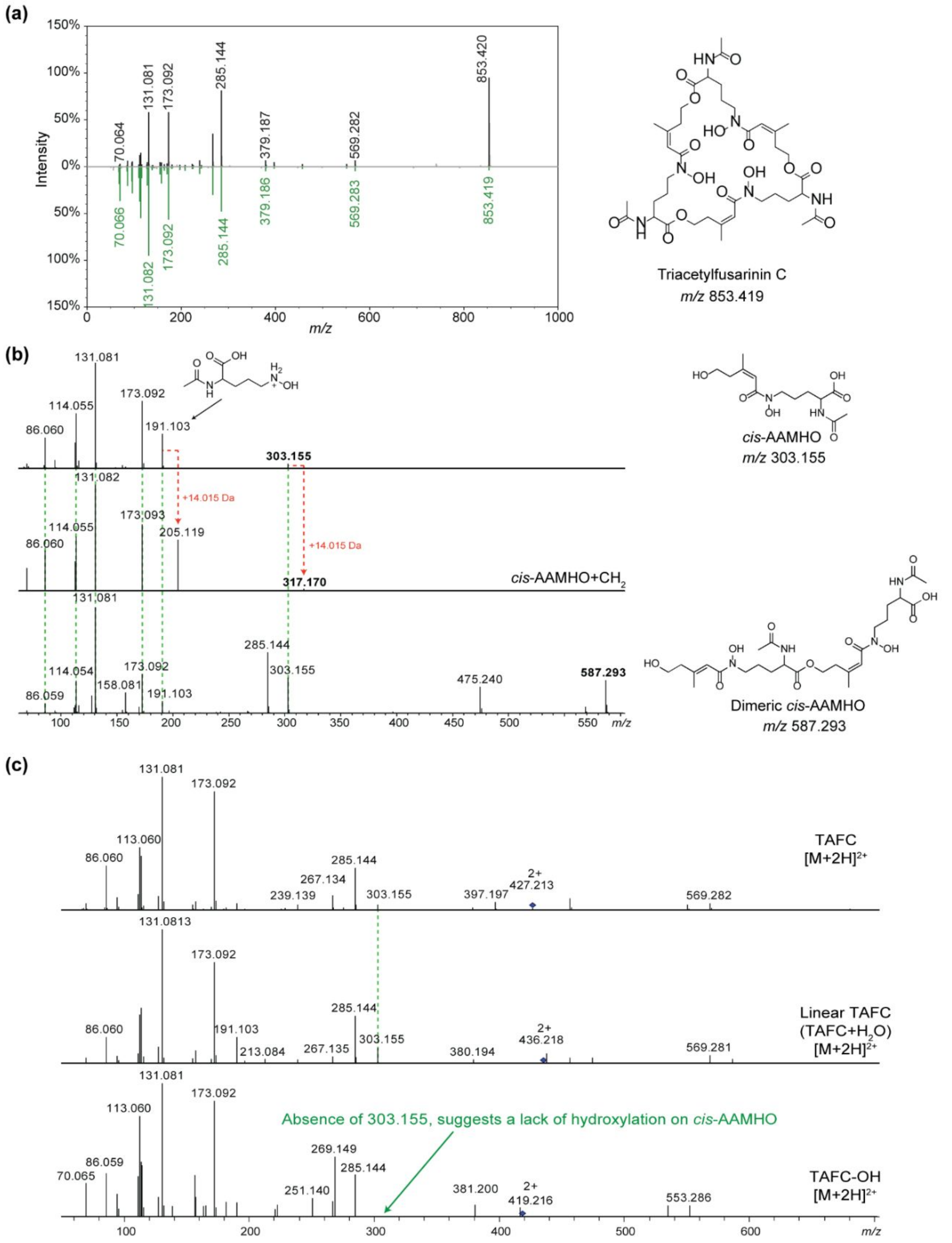

Supplementary Figure S3. (a) Mirror plots comparing experiment MS^2 spectrum of triacetylfusarinin C with published spectrum.[2] (b) MS^2 spectra of the biosynthetic intermediates of TAFC (N_2-acetyl-N_5-cis-anhydromevalonyl-N_5-hydroxy-L-ornithine (*cis*-AAMHO, *m/z* 303.155) and dimeric *cis*-AAMHO (*m/z* 587.293). An analog of *cis*-AAMHO (*m/z* 317.170) consists of an extra methylene group. (c) MS^2 spectra of additional features related to TAFC is shown.

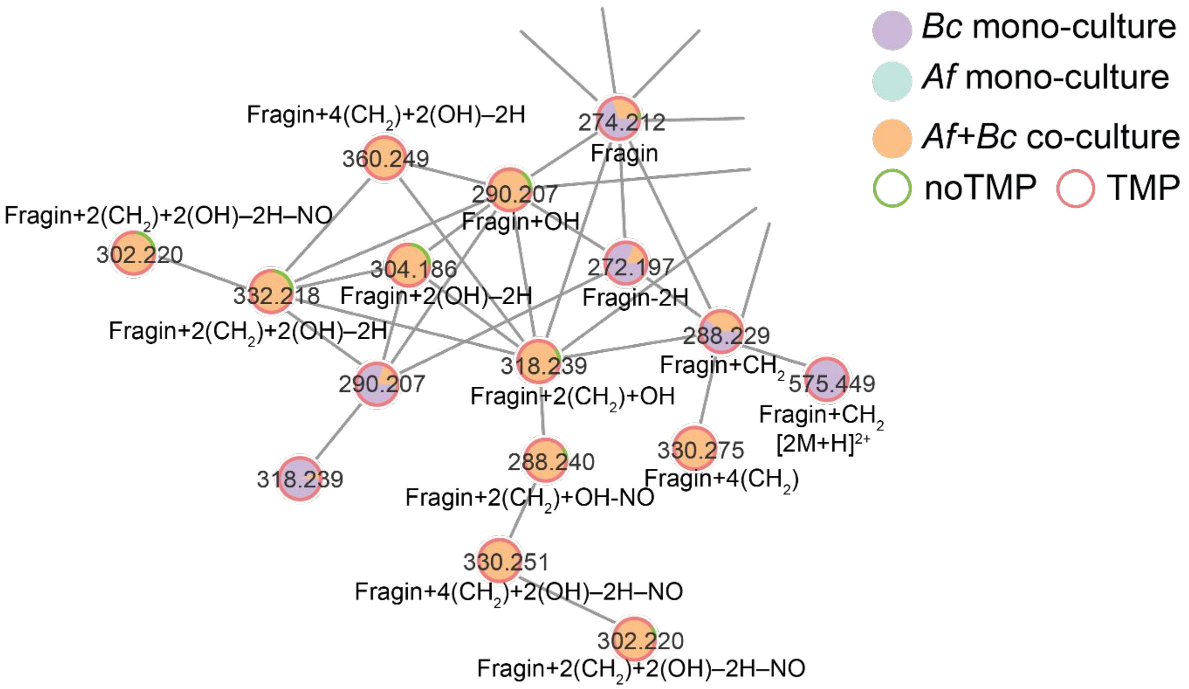

Supplementary Figure S4. Feature-based molecular network hydroxylated and longer chain unsaturated acyl tail-containing fragin analogs. For the sake of clarity, only a portion of the relevant fragin cluster is shown.

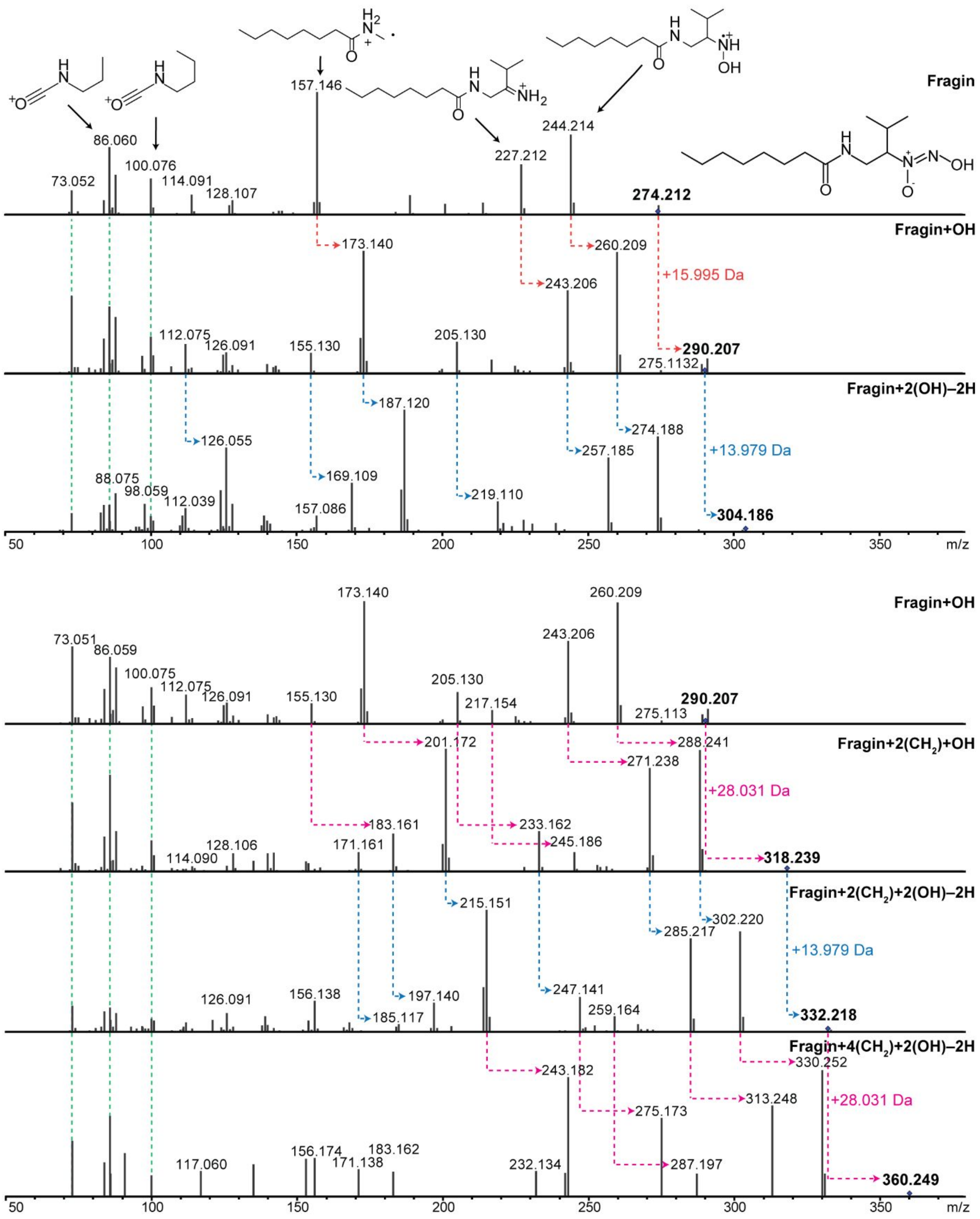

Supplementary Figure S5. MS^2 spectra of fragin and the hydroxylated and longer chain unsaturated acyl tail-containing fragin analogs fragin analogs detected in this study. For each of these features, a peak with neutral loss of 29.998 Da, typical of NO loss for fragin was observed supporting the annotations of these features as fragin analogs.

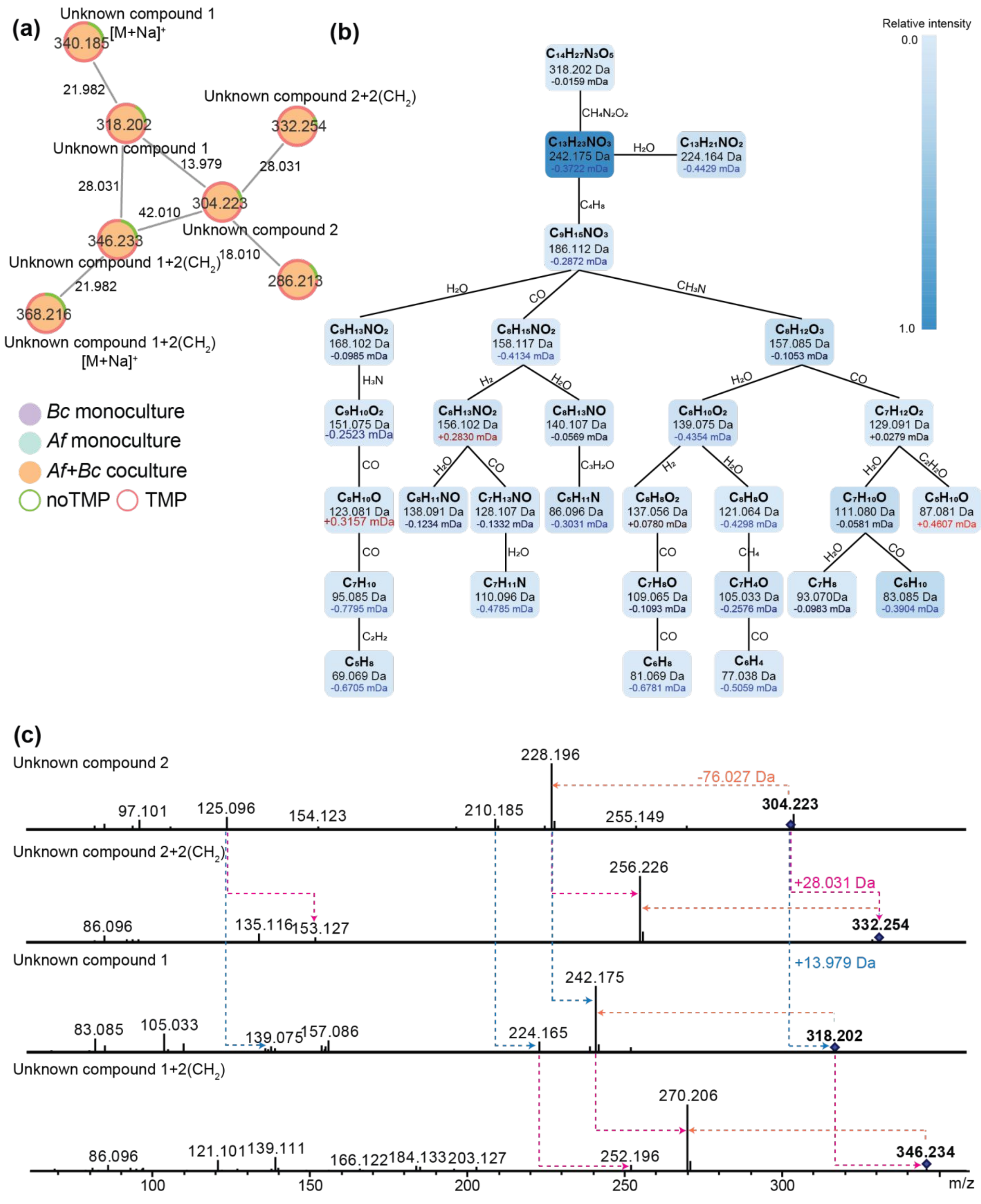

Supplementary Figure S6. A cluster of unknown metabolite features was uniquely detected in the extracts derived from in *Af*+*Bc* co-cultures. (a) Molecular network of the metabolites. (b) Fragmentation tree predicted by SIRIUS based on the MS^2 spectra.[3] These compounds were classified as dipeptides as the tree displays multiple losses of CO, commonly observed in peptide spectra. (c) Alignment of the MS^2 spectra of the unknown compounds for comparison.

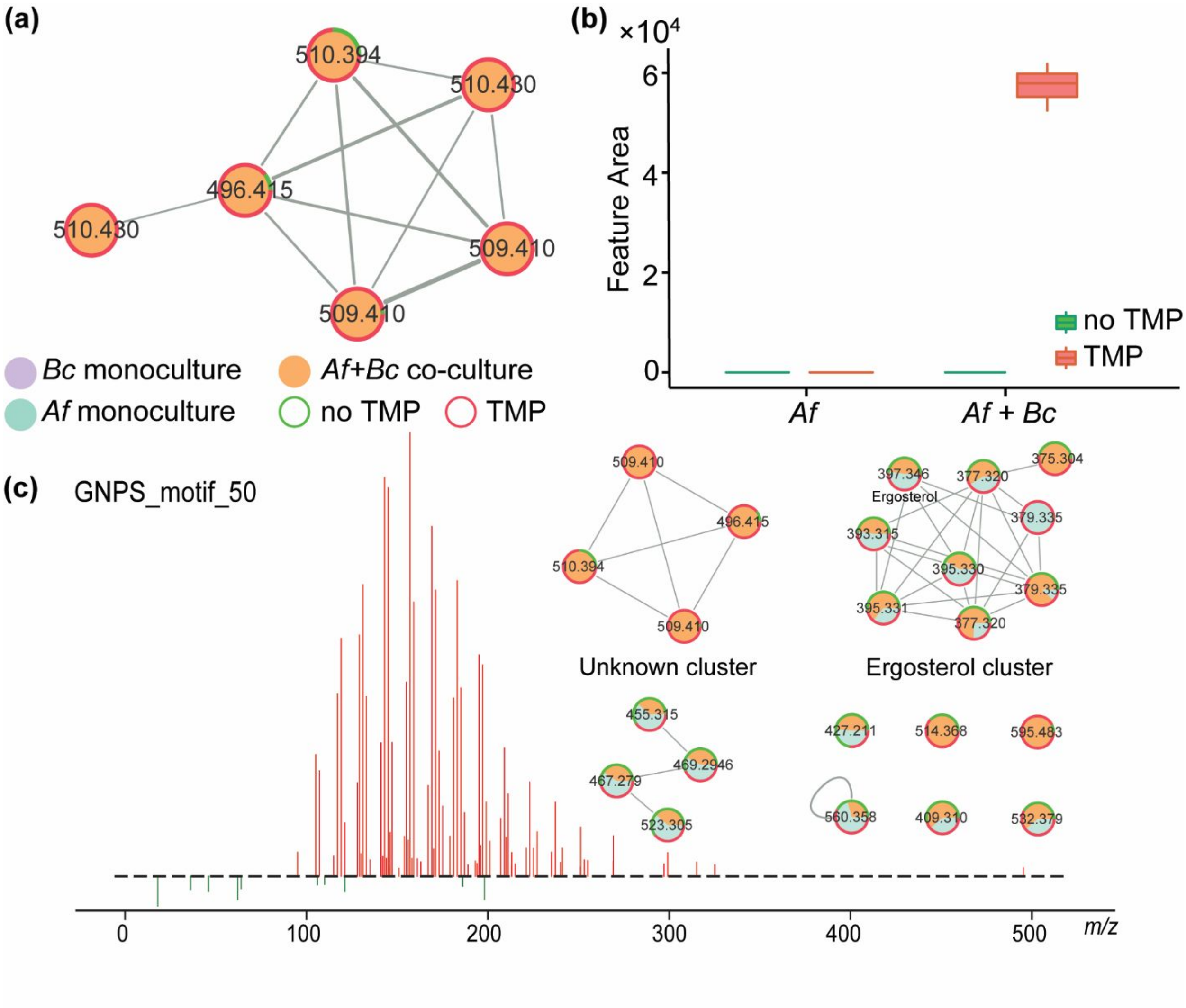

Supplementary Figure S7. Analysis of ergosterol related compounds. (a) Molecular network of unknown features. (b) Box plots of the relative abundance of the feature with *m/z* 509.410. (c) MS2LDA analysis reveals that the unknown cluster and the features in the ergosterol network share the common Mass2Motif GNPS_motif_50.

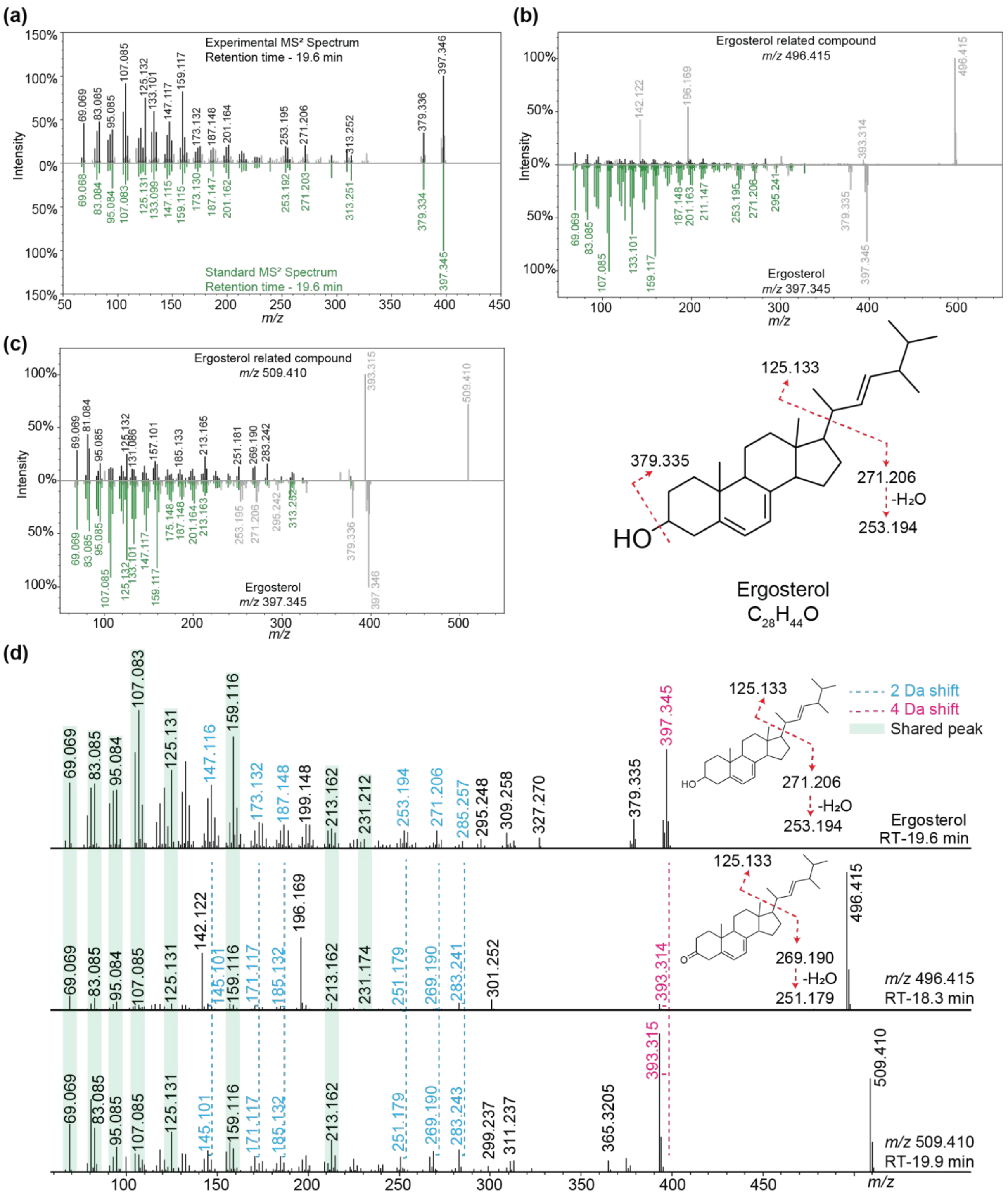

Supplementary Figure S8. Comparative analysis of MS^2 spectra of ergosterol detected in extracts derived from *A. fumigatus* cultures, of commercial standard and related compounds. (a) Mirror plots comparing experimental MS^2 spectrum of ergosterol (top spectrum, black) and MS^2 spectrum of commercial ergosterol standard (bottom spectra, green). (b) Mirror plot comparing spectrum of unknown ergosterol related compound (*m/z* 496.415) (top spectrum, black) with ergosterol (bottom spectra, green). (c) Mirror plot comparing spectrum of unknown ergosterol related compound (*m/z* 509.410) (top spectrum, black) with

ergosterol (bottom spectra, green). (d) MS^2 comparison of analytical standard of ergosterol and the unknown features with *m/z* 496.415 and 509.410 Da.

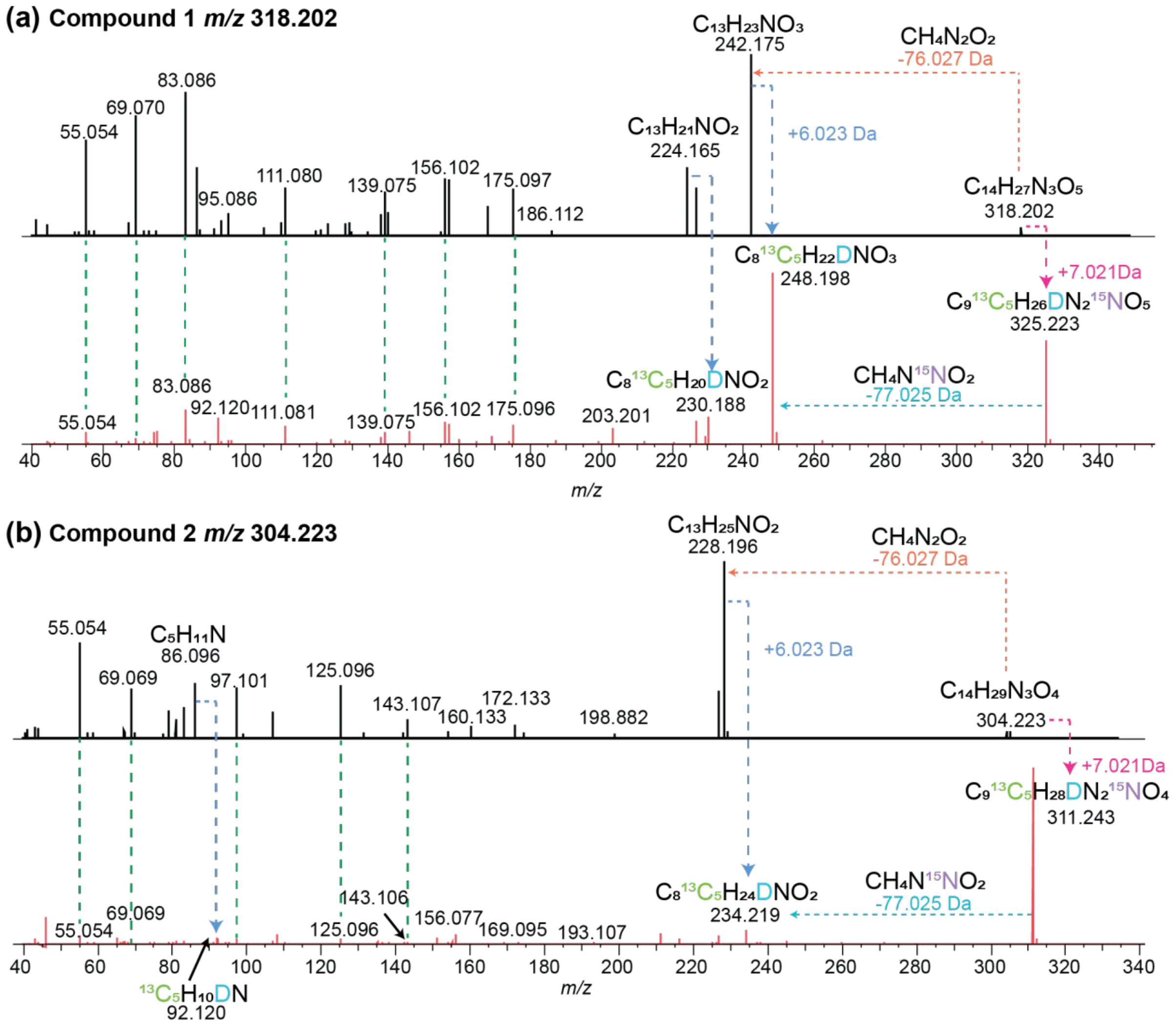

Supplementary Figure S9. MAS-SILAC guided annotation of the fragin related features. Comparative analysis of MS^2 spectra of unlabeled and labeled (via incorporation of with $^{13}C_5$, ^{15}N, D_2-valine) (a) compound 1 (*m/z* 318.202) and (b) compound 2 (*m/z* 304.223).

(a)

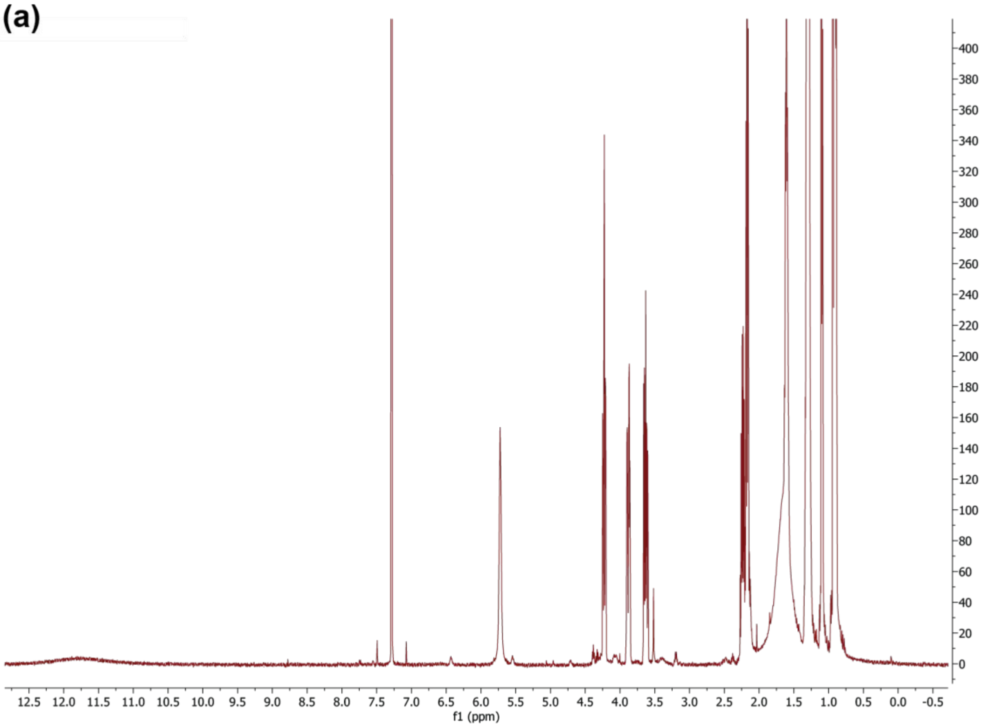

(b) ^{1}H NMR spectrum of fragin in $CDCl_3$(500 MHz, *J* in Hz, *δ* in ppm)

No.	$\delta_{H,}$ multiplicity (*J*, Hz)	
	Jenul et al., 2018	This study
1+1'	0.90, d (6.7)	0.93, d (6.7)
	1.07, d (6.8)	1.09, d (6.9)
2	2.25-2.17, m	2.27-2.19, m
3	4.20, td (9.2, 3.1)	4.23,td (9.2, 3.1)
NOH	11.72, s	11.79, broad
4	3.86, ddd (14.4, 6.0, 3.1)	3.88, ddd (14.4, 6.0, 3.1)
	3.60, ddd (14.4, 9.4, 6.1)	3.63, ddd (14.4, 9.3, 6.1)
NH	5.69, s	5.72, s
5	-	-
6	2.14, td (7.4, 1.8)	2.17, td (7.3, 1.8)
7	1.61-1.52, m	1.63-1.57, m
8-11	1.31-1.23, m	1.33-1.25, m
12	0.87, t (6.8)	0.90, t (6.8)

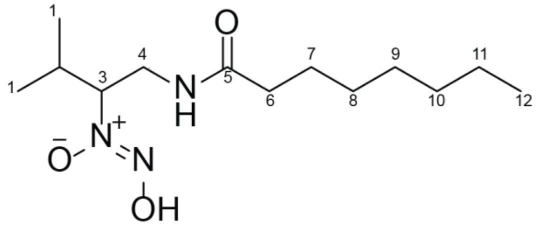

Supplementary Figure S10. Structural analysis of purified fragin. a) ^{1}H NMR spectrum for purified fragin in $CDCl_3$. b) Summary of ^{1}H NMR spectra and comparison to data from Jenul et al., 2018.[4]

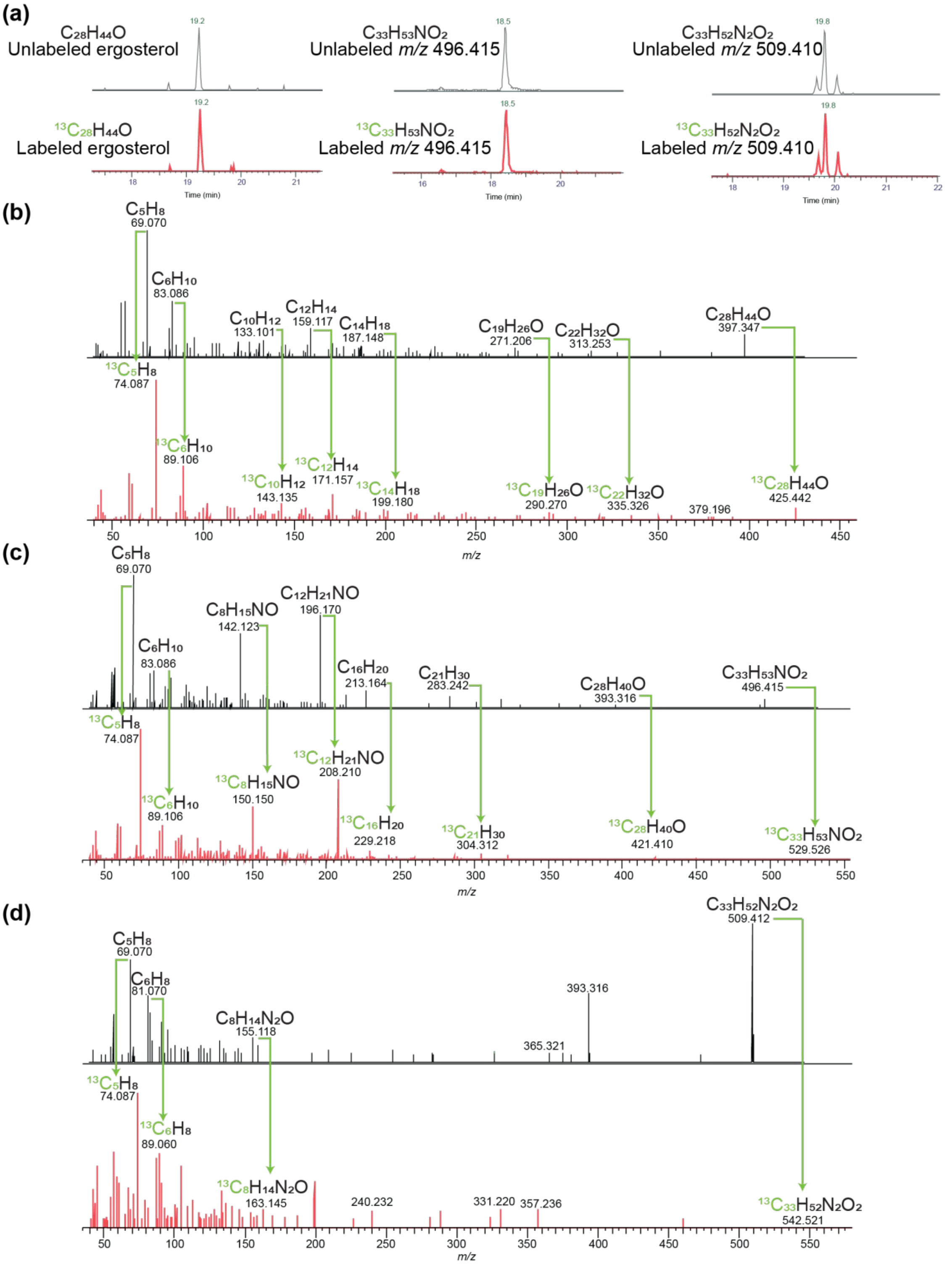

Supplementary Figure S11. Analysis of $^{13}C_6$-glucose labeled ergosterol and related compounds. a) Comparison of EIC of unlabeled and labeled ergosterol (left), unlabeled and labeled compound with *m/z* 496.415 (middle), and unlabeled and labeled compound with *m/z* 509.410 (right). (b) MS^2 spectral analysis of unlabeled and labeled ergosterol, (c) MS^2 spectral analysis of unlabeled and labeled compound with *m/z* 496.415, and (d) MS^2 spectral analysis of unlabeled and labeled compound with *m/z* 509.410.

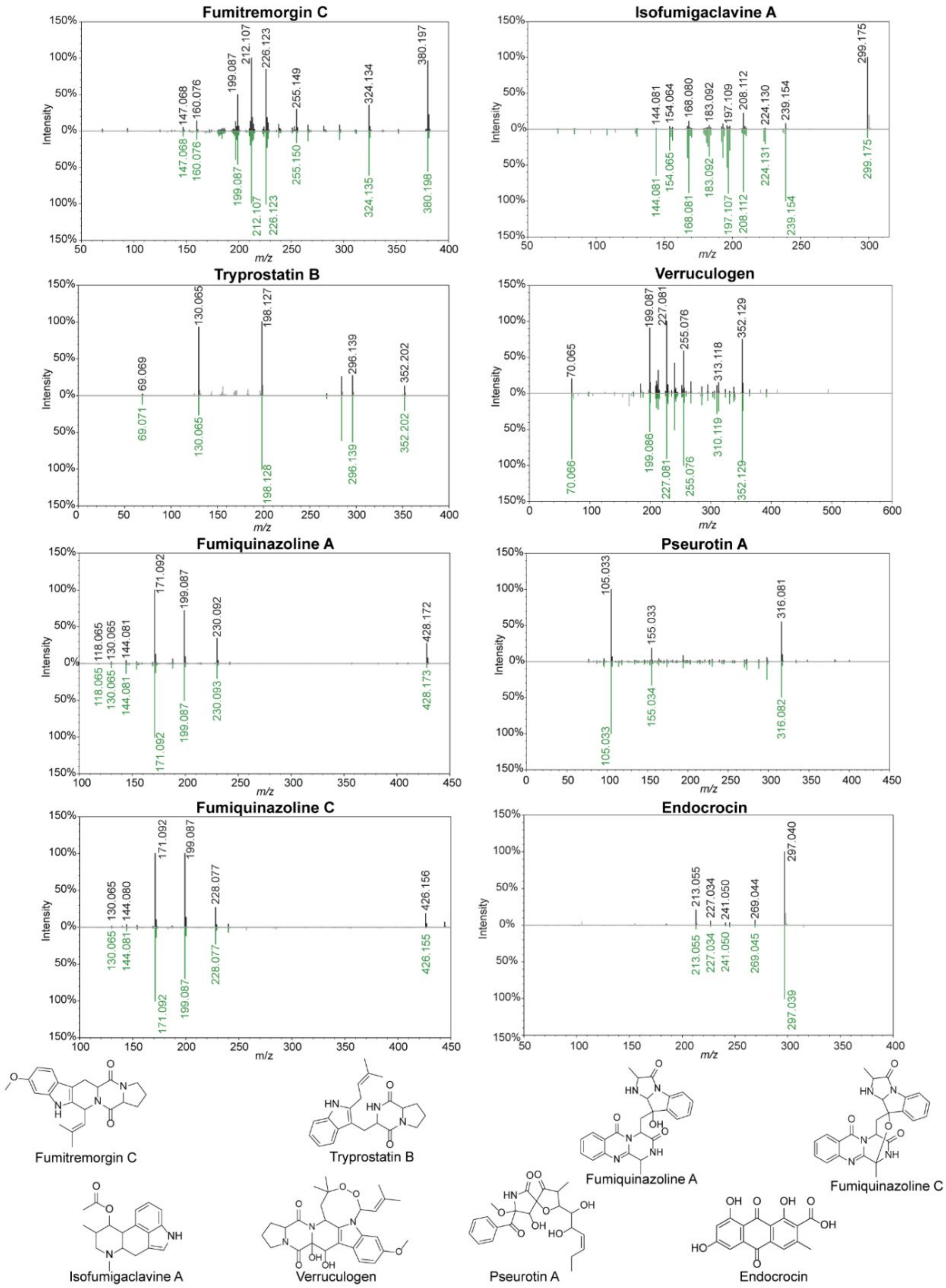

Supplementary Figure S12. Mirror plots comparing experiment MS^2 spectra of mycotoxins detected in this study with spectra deposited in published literature and the GNPS library.[2] Mirror plots were generated with the Metabolomics Spectrum Resolver Web Service.[5]

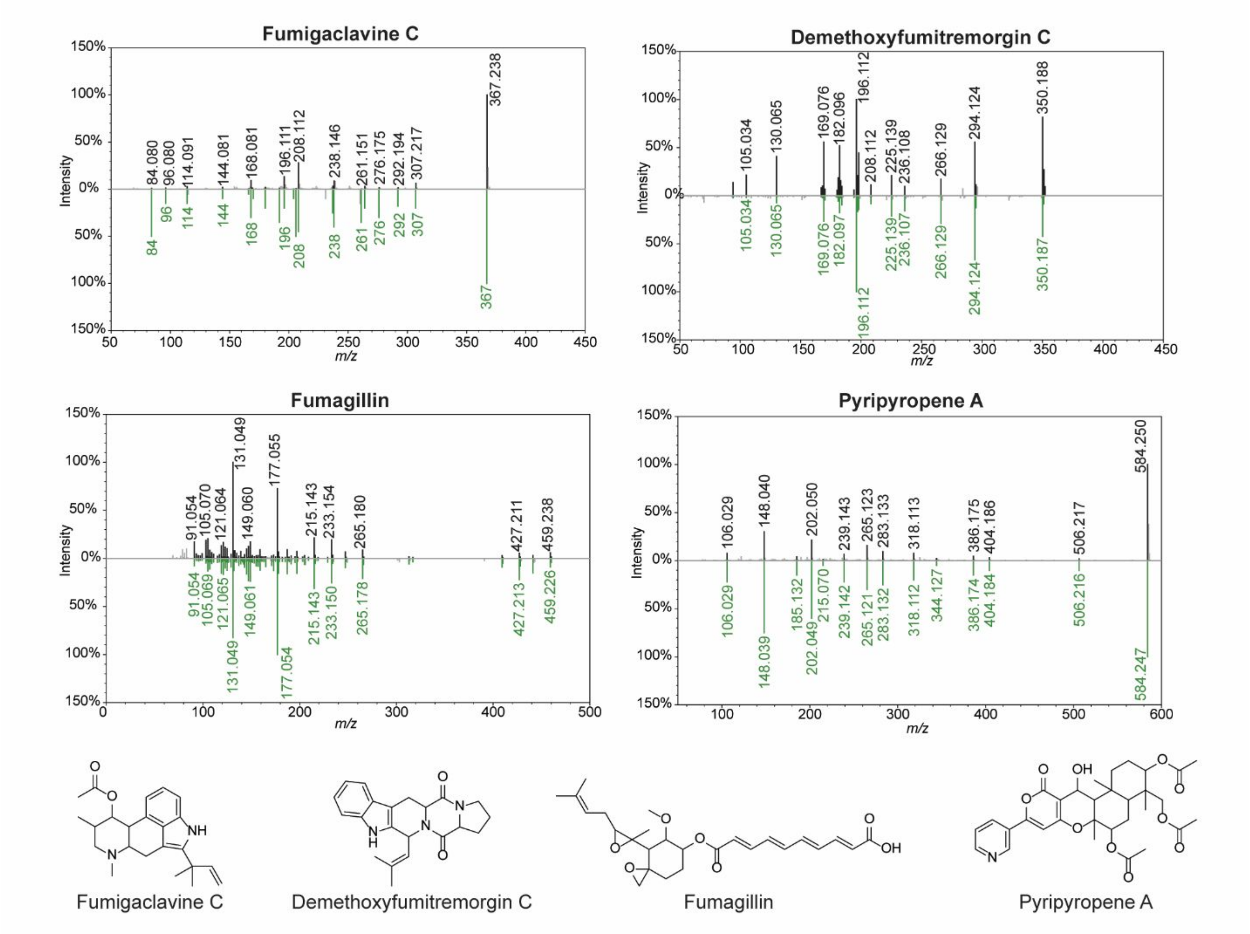

Supplementary Figure S13. Mirror plots comparing experiment MS^2 spectra of mycotoxins detected in this study with spectra published in literature.[6,7] Mirror plots were generated with the Metabolomics Spectrum Resolver Web Service.[5]

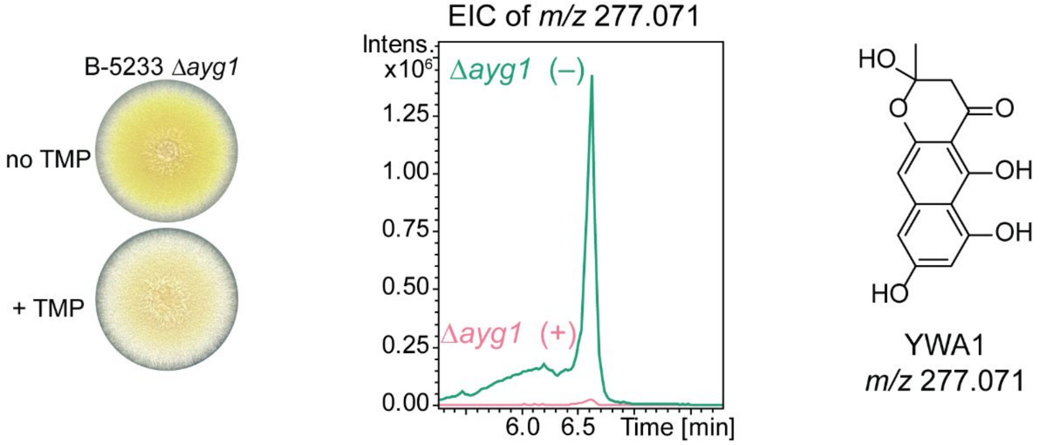

Supplementary Figure S14. Extracted ion chromatogram (EIC) of YWA1 (*m/z* 277.071) detected in extracts derived from monoculture of *A. fumigatus* B-5233 Δ*ayg1* in the presence (**pink +**) of or absence of trimethoprim (**green −**).

Supplementary Table S1. Bacterial and fungal strains, plasmids, and primers used in this study.

Strains	Description	Source
Burkholderia cenocepacia		
K56-2	CF isolate, $BCESM^{+}$*cbl*A^{+}	[8]
K56-2 Δ*hamC*	Δ*hamC* (BCAM0194) deletion mutant	This study
Aspergillus fumigatus		
A1160+	*A. fumigatus* strain derived from CEA10 that lacks nonhomologous recombination. $\Delta akuB^{ku80}::pyrG^{-}zeo$, $pyrG^{-}::pyrG^{Af}$; *MAT1-1*	[9, 10]
B-5233	A clinical isolate strain from a patient with a fatal invasive aspergillosis	[11]
B-5233 Δ*alb1*	an *alb1* gene deletant of B-5233	[11]
B-5233 Δ*ayg1*	an *ayg1* gene deletant of B-5233	[11]
B-5233 Δ*arp2*	an *arp2* gene deletant of B-5233	[11]
B-5233 Δ*arp1*	an *arp1* gene deletant of B-5233	[12]
B-5233 Δ*abr1*	an *abr1* gene deletant of B-5233	[11]
B-5233 Δ*abr2*	an *abr2* gene deletant of B-5233	[11]
Escherichia coli		
DH5α	*fhuA2 lac(del)U169 phoA glnV44 Φ80' lacZ(del)M15 gyrA96 recA1 relA1 endA1 thi-1 hsdR17*	
SM10	*thi thr leu tonA lacY supE recA::RP4-2-Tc::Mu Km λpir*	

Plasmids	Description	Source
pMo130	*Burkholderia* suicide vector Km^{R}	[13]
pMo130-Tc	Km^{R} cassette replaced with Tc^{R}	This study
pMo130-Tc_Δ*hamC*	*hamC* deletion vector	This study

Primers	Name	Sequence
P1	hamC_up_F	TATCCCCTGATTCTGTGGATGTGAATCAACTGGAAGGGCTGGAGCGA
P2	hamC_up_R	GTAGTCGGACAACACGTAGGTGACGGCGGTC
P3	hamC_down_F	GTCACCTACGTGTTGTCCGACTACCTGTGAGTC
P4	hamC_down_R	CTCACTCAAAGGCGGTAATACGGTTGATCGCGACCGCGCAGC
P5	hamC_del_F	CGACCTATCGGGAAGGTAGC
P6	hamC_del_R	TATGACCGGTCGCCTCGAG
P7	hamC_seq_F	AGCCCGATTTGCGGATGTCAATTC
P8	hamC_seq_R	ATTCGGTCGGCCCGTAGATGTTG

Supplementary Table S2. Putative annotation of top 50 metabolite features detected in Heatmap.

m/z_RT	Formula	Adduct	Annotation	Source
351.178_15.5	$C_{17}H_{28}O_6$	$[M+Na]^+$	-	Sirius
485.326_11.2	$C_{26}H_{40}N_6O_3$	$[M+H]^+$	Steroids and steroid derivatives	CANOPUS
509.276_9.0	$C_{28}H_{36}N_4O_5$	$[M+H]^+$	Oligopeptides	CANOPUS
395.330_19.6	$C_{28}H_{42}O$	$[M+H]^+$	Dehydroergosterol	FBMN
560.358_15.3	$C_{32}H_{49}NO_7$	$[M+H]^+$	Steroid esters	CANOPUS
542.348_15.3	$C_{32}H_{47}NO_6$	$[M+H]^+$	Steroid esters	CANOPUS
253.195_19.6	$C_{19}H_{24}$	$[M+H]^+$	Steroids and steroid derivatives	CANOPUS
504.368_19.6	$C_{30}H_{46}O_5$	$[M+NH_4]^+$	Steroids and steroid derivatives	CANOPUS
219.138_9.4	$C_{14}H_{18}O_2$	$[M+H]^+$	Indanones	CANOPUS
249.110_14.3	$C_{12}H_{18}O_4$	$[M+Na]^+$	-	Sirius
234.134_1.2	$C_{11}H_{15}N_5O$	$[M+H]^+$	6-alkylaminopurines	CANOPUS
273.160_4.8	$C_{16}H_{20}N_2O_2$	$[M+H]^+$	Indoles	CANOPUS
179.112_7.5	$C_5H_{16}N_4O_4$	$[M–H_2O+H]^+$	1,2-aminoalcohols	CANOPUS
163.117_7.2	-	-	-	-
357.217_7.2	$C_{21}H_{28}N_2O_3$	$[M+H]^+$	Carbazoles	CANOPUS
257.165_7.2	$C_{16}H_{20}N_2O$	$[M+H]^+$	Fumigaclavine B	MS2LDA and CMM
273.159_7.9	$C_{16}H_{20}N_2O_2$	$[M+H]^+$	Quinolines and derivatives	CANOPUS
260.152_7.9	$C_{13}H_{19}N_5O_2$	$[M–H_2O+H]^+$	Azoles	CANOPUS
187.110_7.9	-	-	-	-
359.232_7.9	$C_{21}H_{30}N_2O_3$	$[M+H]^+$	Quinolizidines	CANOPUS
373.212_7.9	$C_{21}H_{28}N_2O_4$	$[M+H]^+$	Aspidospermatan-type alkaloids	CANOPUS
341.222_7.9	$C_{21}H_{28}N_2O_2$	$[M+H]^+$	3-alkylindoles	CANOPUS
170.154_1.2	$C_{10}H_{19}NO$	$[M+H]^+$	Amines	CANOPUS
186.102_7.7	-	-	-	-
331.165_7.7	$C_{18}H_{22}N_2O_4$	$[M+H]^+$	Benzenoids	CANOPUS
355.202_13.1	$C_{21}H_{26}N_2O_3$	$[M+H]^+$	Amino acids and derivatives	CANOPUS
312.152_4.7	$C_{12}H_{23}N_3O_5$	$[M+Na]^+$	Alpha amino acid amides	CANOPUS
290.207_5.6	$C_{13}H_{27}N_3O_4$	$[M+H]^+$	Fragin+OH	FBMN
318.202_5.9	$C_{14}H_{27}N_3O_5$	$[M+H]^+$	Unknown compound 1	CANOPUS
496.415_18.3	$C_{33}H_{53}NO_2$	$[M+H]^+$	Ergosterol+C_5H_9NO	MS2LDA
304.223_6.0	$C_{14}H_{29}N_3O_4$	$[M+H]^+$	Unknown compound 2	CANOPUS
288.240_7.3	$C_{15}H_{31}N_2O_3$	$[M+H]^+$	Fragin+2(CH_2)+OH–NO	FBMN
318.238_7.3	$C_{15}H_{31}N_3O_4$	$[M+H]^+$	Fragin+2(CH_2)+OH	FBMN
242.174_5.9	$C_{13}H_{25}NO_4$	$[M–H_2O+H]^+$	Amino acids and derivatives	CANOPUS
326.169_5.5	$C_{13}H_{25}N_3O_5$	$[M+Na]^+$	Fragin+2(OH)–2H	FBMN
368.216_7.4	$C_{16}H_{31}N_3O_5$	$[M+Na]^+$	Unknown compound 1+2(CH_2)	FBMN
354.200_7.2	$C_{15}H_{29}N_3O_5$	$[M+Na]^+$	Fragin+2(CH_2)+2(OH)–2H	MS2 similarity
332.218_7.2	$C_{15}H_{29}N_3O_5$	$[M+H]^+$	Fragin+2(CH_2)+2(OH)–2H	FBMN
340.185_5.9	$C_{14}H_{27}N_3O_5$	$[M+Na]^+$	Unknown compound 1	FBMN
302.220_7.2	$C_{15}H_{29}N_2O_4$	$[M+H]^+$	Fragin+2(CH_2)+2(OH)–2H–NO	FBMN
270.206_7.4	$C_{15}H_{27}NO_3$	$[M+H]^+$	N-acylpyrrolidines	CANOPUS
346.233_7.4	$C_{16}H_{31}N_3O_5$	$[M+H]^+$	Unknown compound 1+2(CH_2)	FBMN

244.215_9.3	$C_{13}H_{27}N_2O_2$	$[M+H]^+$	Fragin–NO	FBMN
178.117_11.2	-	-	-	-
270.230_10.4	$C_{15}H_{29}N_2O_2$	$[M+H]^+$	Fragin+2(CH_2)–NO–2H	FBMN
302.244_11.2	$C_{15}H_{31}N_3O_3$	$[M+H]^+$	Fragin+2(CH_2)	FBMN
250.112_7.3	$C_{11}H_{17}N_5OS$	$[M–H_2O+H]^+$	6-thiopurines	CANOUPS
218.140_4.1	$C_{11}H_{17}N_5O$	$[M–H_2O+H]^+$	6-alkylaminopurines	CANOUPS
275.161_7.8	$C_{15}H_{24}O_3$	$[M+Na]^+$	-	SIRIUS
537.499_19.1	$C_{33}H_{64}N_2O_3$	$[M+H]^+$	N-acyl amines	CANOPUS

Supplementary Table S3. List of metabolite features detected exclusively in co-cultures in UpSet plot.

**155 features detected exclusively in co-culture conditions**				
*m/z*_RT	Formula	Adduct	Annotation / compound class	Source
318.202_5.9	$C_{14}H_{27}N_3O_5$	$[M+H]^+$	Unknown compound 1	CANOPUS
302.220_7.2	$C_{16}H_{29}N_3O$	$[M+Na]^+$	Fragin+2(CH_2)+(2OH)–2H–NO	FBMN
332.218_7.2	$C_{15}H_{29}N_3O_5$	$[M+H]^+$	Fragin+2(CH_2)+2(OH)–2H	FBMN
354.200_7.2	$C_{15}H_{29}N_3O_5$	$[M+Na]^+$	Fragin+2(CH_2)+2(OH)–2H	FBMN
853.419_6.4	$C_{39}H_{60}N_6O_{15}$	$[M+H]^+$	Triacetylfusarinin C (TAFC)	GNPS Library
346.233_7.4	$C_{16}H_{31}N_3O_5$	$[M+H]^+$	Unknown compound 1+2(CH_2)	CANOPUS
368.216_7.4	$C_{16}H_{31}N_3O_5$	$[M+Na]^+$	Unknown compound 1+2(CH_2)	CANOPUS
304.186_5.6	$C_{13}H_{25}N_3O_5$	$[M+H]^+$	Amino acids and derivatives	CANOPUS
326.169_5.5	$C_{13}H_{25}N_3O_5$	$[M+Na]^+$	Amino acids and derivatives	FBMN
453.669_6.1	$C_{39}H_{57}FeN_6O_{15}$	$[M+2H]^{2+}$	TAFC-iron complex	CMM
496.415_18.3	$C_{33}H_{53}NO_2$	$[M+H]^+$	Ergosterol+C_5H_9NO	MS2LDA
304.223_6.0	$C_{14}H_{29}N_3O_4$	$[M+H]^+$	Unknwon dipeptide 1	CANOPUS
875.401_6.4	$C_{39}H_{60}N_6O_{15}$	$[M+Na]^+$	TAFC	CMM
288.216_6.4	$C_{15}H_{31}NO_5$	$[M–H_2O+H]^+$	Amino acids and derivatives	CANOPUS
226.115_3.2	$C_8H_{17}N_3O_3$	$[M+Na]^+$	Amino acids and derivatives	CANOPUS
492.441_19.1	$C_{31}H_{57}NO_3$	$[M+H]^+$	N-acyl amines	CANOPUS
270.206_7.4	$C_{15}H_{27}NO_3$	$[M+H]^+$	N-acylpyrrolidines	CANOPUS
271.238_7.3	$C_{15}H_{30}N_2O_2$	$[M+H]^+$	Alpha amino acids and derivatives	CANOPUS
256.227_7.3	$C_{15}H_{31}NO_3$	$[M–H_2O+H]^+$	1,2-aminoalcohols	CANOPUS
326.205_6.0	$C_{14}H_{29}N_3O_4$	$[M+Na]^+$	Amino acids and derivatives	CANOPUS
443.224_6.4	$C_{40}H_{64}N_6O_{16}$	$[M+2H]^{2+}$	TAFC+CH_2+H_2O	FBMN
270.206_8.1	$C_{15}H_{27}NO_3$	$[M+H]^+$	N-acylpyrrolidines	CANOPUS
514.423_19.1	$C_{27}H_{53}N_7O$	$[M+Na]^+$	Heteroaromatic compounds	CANOPUS
282.178_7.2	$C_{12}H_{25}N_3O_3$	$[M+Na]^+$	Alpha amino acid amides	CANOPUS
256.227_8.3	$C_{15}H_{31}NO_3$	$[M–H_2O+H]^+$	Carboxylic acid amides	CANOPUS
426.321_12.3	$C_{24}H_{40}O_5$	$[M+NH_4]^+$	Diterpenoids	CANOPUS
274.189_5.7	$C_{11}H_{23}N_5O_3$	$[M+H]^+$	Amino acids and derivatives	CANOPUS
402.298_16.4	$C_{23}H_{41}NO_3$	$[M+Na]^+$	-	SIRIUS
439.194_5.7	-	$[M+2H]^{2+}$	TAFC analog	FBMN
296.170_5.6	$C_{11}H_{23}N_5O_3$	$[M+Na]^+$	Alpha amino acid esters	CANOPUS
284.122_3.6	$C_{10}H_{19}N_3O_5$	$[M+Na]^+$	-	SIRIUS
446.186_6.4	-	$[M+2H]^{2+}$	TAFC analog	FBMN
286.212_9.2	$C_{14}H_{27}N_3O_3$	$[M+H]^+$	Amino acids and derivatives	CANOPUS
260.197_7.2	$C_{12}H_{25}N_3O_3$	$[M+H]^+$	Fatty Acyls	CANOPUS
928.312_6.1	$C_{39}H_{57}FeN_6O_{15}$	$[M+Na]^+$	TAFC-iron complex	CMM
336.178_8.7	$C_{16}H_{23}N_5O$	$[M+Na]^+$	Pyrrolidinecarboxamides	CANOPUS
308.194_9.0	$C_{14}H_{27}N_3O_3$	$[M+Na]^+$	Amino acids, peptides, and analogs	CANOPUS
191.103_2.0	$C_7H_{14}N_2O_4$	$[M+H]^+$	*N*5-acetyl-*N*5-hydroxy-L-ornithine	MS2LDA
404.313_17.4	$C_{23}H_{43}NO_3$	$[M+Na]^+$	-	SIRIUS

354.235_7.5	$C_{16}H_{33}N_3O_4$	$[M+Na]^+$	Amino acids and derivatives	CANOPUS
347.190_2.6	$C_{14}H_{28}N_4O_7$	$[M–H_2O+H]^+$	Peptides	CANOPUS
436.218_6.0	$C_{39}H_{62}N_6O_{16}$	$[M+2H]^{2+}$	TAFC+H_2O	FBMN
378.298_17.2	$C_{21}H_{41}NO_3$	$[M+Na]^+$	N-acyl-alpha amino acids and derivatives	CANOPUS
560.358_15.7	$C_{32}H_{47}NO_6$	$[M+H_2O+H]^+$	Steroid esters	CANOPUS
563.265_14.8	$C_{34}H_{34}N_4O_4$	$[M+H]^+$	Porphyrins	CANOPUS
302.220_7.8	$C_{18}H_{29}N_3O_2$	$[M–H_2O+H]$	N-alkylpiperazines	FBMN
332.254_7.5	$C_{16}H_{33}N_3O_4$	$[M+H]^+$	N-acyl-alpha amino acids and derivatives	CANOPUS
398.290_11.3	$C_{22}H_{36}O_5$	$[M+NH_4]^+$	Dicarboxylic acids and derivatives	CANOPUS
360.217_9.4	$C_{21}H_{29}NO_4$	$[M+H]^+$	N-acyl amines	CANOPUS
353.305_12.5	$C_{22}H_{42}O_4$	$[M–H_2O+H]^+$	1-monoacylglycerols	CANOPUS
583.415_9.3	$C_{29}H_{54}N_6O_6$	$[M+H]^+$	Dipeptides	CANOPUS
760.608_22.7	$C_{46}H_{81}NO_7$	$[M+H]^+$	Triacylglycerols	CANOPUS
434.221_6.7	$C_{40}H_{62}N_6O_{15}$	$[M+2H]^{2+}$	TAFC+CH_2	FBMN
384.310_19.1	$C_{22}H_{41}NO_3$	$[M+O+H]$	N-acyl amines	CANOPUS
906.330_6.1	$C_{39}H_{57}FeN_6O_{15}$	$[M+H]^+$	TAFC-iron complex	CMM
230.211_19.1	$C_{13}H_{29}NO_3$	$[M–H_2O+H]^+$	Quaternary ammonium salts	CANOPUS
857.454_9.2	$C_{44}H_{64}N_4O_{13}$	$[M+H]^+$	1-hydroxysteroids	CANOPUS
595.483_19.7	$C_{43}H_{62}O$	$[M+H]^+$	Ergosterol related	MS2LDA
336.159_10.7	$C_{17}H_{17}N_7O$	$[M+H]^+$	Aminopyrimidines and derivatives	CANOPUS
419.216_6.4	$C_{39}H_{59}N_6O_{14}$	$[M+2H]^{2+}$	TAFC-OH	FBMN
448.303_12.3	$C_{24}H_{43}NO_5$	$[M+Na]^+$	Fatty acid esters	CANOPUS
326.184_9.4	$C_{14}H_{29}N_3O_3$	$[M+K]^+$	-	SIRIUS
537.499_19.1	$C_{33}H_{66}N_2O_4$	$[M–H_2O+H]^+$	Amino acids and derivatives	CANOPUS
358.202_8.9	$C_{21}H_{27}NO_4$	$[M+H]^+$	Isoindolones	CANOPUS
539.514_19.8	$C_{33}H_{66}N_2O_3$	$[M+H]^+$	N-acyl amines	CANOPUS
380.315_17.2	$C_{23}H_{41}NO_3$	$[M+H]^+$	N-acyl-alpha amino acids	CANOPUS
226.113_1.9	$C_8H_{17}N_3O_3$	$[M+Na]^+$	Alpha amino acids and derivatives	CANOPUS
342.206_10.9	$C_{21}H_{27}NO_3$	$[M+H]^+$	Benzene and subsituted derivatives	CANOPUS
472.642_6.1	$C_{39}H_{57}FeN_6O_{16}$	$[M+H+K]^{2+}$	TAFC-iron complex	FBMN
312.362_15.3	$C_{21}H_{45}N$	$[M+H]^+$	-	SIRIUS
242.175_7.4	$C_{13}H_{25}NO_4$	$[M–H_2O+H]^+$	Alpha amino acid esters	CANOPUS
509.410_19.6	$C_{33}H_{52}N_2O_2$	$[M+H]^+$	Ergosterol+ $C_5H_8N_2O$	MS2LDA
368.215_8.0	$C_{25}H_{25}N_3$	$[M+H]^+$	-	SIRIUS
520.472_19.9	$C_{33}H_{61}NO_3$	$[M+H]^+$	N-acyl amines	CANOPUS
353.305_12.8	$C_{22}H_{42}O_4$	$[M–H_2O+H]^+$	1-monoacylglycerols	CANOPUS
325.274_12.1	$C_{20}H_{36}O_3$	$[M+H]^+$	-	SIRIUS
375.304_15.8	$C_{28}H_{38}$	$[M+H]^+$	Prenol lipids	CANOPUS
358.141_10.7	$C_{21}H_{21}NO_3$	$[M+Na]^+$	-	SIRIUS

597.431_9.4	$C_{33}H_{58}N_4O_4$	$[M+Na]^+$	Alpha amino acids and derivatives	CANOPUS
289.237_9.9	$C_{16}H_{32}O_4$	$[M+H]^+$	Long-chain fatty acids	CANOPUS
446.324_19.1	$C_{25}H_{45}NO_4$	$[M+Na]^+$	Steroids and steroid derivatives	CANOPUS
367.147_4.4	$C_{15}H_{24}N_2O_7$	$[M+Na]^+$	-	SIRIUS
762.624_18.3	$C_{46}H_{83}NO_7$	$[M+H]^+$	N-acyl-alpha amino acids	CANOPUS
406.293_19.1	$C_{22}H_{41}NO_4$	$[M+Na]^+$	-	SIRIUS
334.199_16.4	$C_{19}H_{29}NO_5$	$[M–H_2O+H]^+$	Valine and derivatives	CANOPUS
286.213_8.5	$C_{14}H_{27}N_3O_3$	$[M+H]^+$	Amino acids and derivatives	CANOPUS
483.272_10.8	$C_{22}H_{43}O_9P$	$[M+H]^+$	PG(16:1(9Z)/0:0)	FBMN and CMM
517.468_9.3	$C_{28}H_{60}N_4O_4$	$[M+H]^+$	Amino acids and derivatives	CANOPUS
503.419_19.4	$C_{29}H_{56}N_2O_3$	$[M+Na]^+$	Amino acids and derivatives	CANOPUS
527.418_18.7	$C_{28}H_{51}N_3O_5$	$[M+NH_4]^+$	Amino acids and derivatives	CANOPUS
266.639_12.5	-	-	-	-
803.645_22.0	$C_{55}H_{84}N_2O_3$	$[M–H_2O+H]^+$	N-acyl amines	CANOPUS
345.165_4.4	$C_{15}H_{24}N_2O_7$	$[M+H]^+$	Alpha amino acid esters	CANOPUS
603.453_16.2	$C_{42}H_{60}O$	$[M+Na]^+$	Xanthophylls	FBMN
803.644_21.8	$C_{49}H_{86}O_8$	$[M+H]^+$	Triacylglycerols	CANOPUS
390.298_16.9	$C_{20}H_{35}N_7O$	$[M+H]^+$	-	SIRIUS
512.503_20.8	$C_{32}H_{65}NO_3$	$[M+H]^+$	N-acyl amines	CANOPUS
513.499_19.7	$C_{31}H_{64}N_2O_3$	$[M+H]^+$	N-acyl amines	CANOPUS
263.237_16.4	$C_{18}H_{30}O$	$[M+H]^+$	Fatty acyls	CANOPUS
413.337_9.9	$C_{23}H_{44}N_2O_4$	$[M+H]^+$	N-acyl-alpha amino acids and derivatives	CANOPUS
758.574_16.2	$C_{42}H_{80}NO_8P$	$[M+H]^+$	1-Hexadecanoyl-2-octadecadienoyl-sn-glycero-3-phosphocholine	GNPS library
508.34_12.6	$C_{25}H_{50}NO_7P$	$[M+H]^+$	Amino acids and derivatives	CANOPUS
514.368_20.0	$C_{33}H_{49}NO_2$	$[M+Na]^+$	Ergosterol+C_5H_9NO+H_2O	MS2LDA
542.348_15.7	$C_{32}H_{47}NO_6$	$[M+H]^+$	Steroid esters	CANOPUS
529.434_19.4	$C_{31}H_{58}N_2O_3$	$[M+Na]^+$	Fatty acyls	CANOPUS
352.155_8.8	$C_{21}H_{23}NO_5$	$[M–H_2O+H]^+$	N-acyl-alpha amino acids and derivatives	CANOPUS
510.394_18.8	$C_{33}H_{51}NO_3$	$[M+H]^+$	Ergosterol+$C_5H_7NO_2$	MS2LDA
369.347_13.1	$C_{22}H_{44}N_2O_2$	$[M+H]^+$	Morpholines	CANOPUS
382.331_18.2	$C_{23}H_{43}NO_3$	$[M+H]^+$	N-acyl-alpha amino acids	CANOPUS
738.624_22.6	$C_{44}H_{84}NO_7$	$[M+H]^+$	DGTSA(16:0/18:1)	GNPS library
750.624_22.0	$C_{45}H_{83}NO_7$	$[M+H]^+$	Triacylglycerols	CANOPUS
466.425_18.9	$C_{29}H_{55}NO_3$	$[M+H]^+$	N-acyl amines	CANOPUS
762.624_17.4	$C_{41}H_{83}N_3O_9$	$[M+H]^+$	Alpha amino acid esters	CANOPUS
261.114_10.8	$C_{18}H_{15}NO$	$[M+H]^+$	-	Bruker
803.645_22.6	$C_{49}H_{86}O_8$	$[M+H]^+$	Triacylglycerols	CANOPUS
267.159_10.6	$C_{15}H_{22}O_4$	$[M+H]^+$	Benzoic acid esters	CANOPUS
758.572_16.6	$C_{42}H_{80}NO_8P$	$[M+H]^+$	1-Hexadecanoyl-2-octadecadienoyl-sn-glycero-3-phosphocholine	GNPS library

384.311_19.8	$C_{22}H_{41}NO_3$	$[M+O+H]^+$	N-acyl amines	CANOPUS
496.303_11.1	$C_{23}H_{46}NO_8P$	$[M+H]^+$	Amino acids and derivatives	CANOPUS
275.644_12.7	-	-	-	-
272.186_9.4	$C_{15}H_{21}N_5$	$[M+H]^+$	Dialkylarylamines	FBMN
265.252_17.4	$C_{18}H_{34}O_2$	$[M–H_2O+H]^+$	Long-chain fatty acids	CANOPUS
279.165_1.8	$C_{10}H_{22}N_4O_5$	$[M+H]^+$	Alpha amino acids and derivatives	CANOPUS
242.175_7.7	$C_{13}H_{23}NO_3$	$[M+H]^+$	Alpha amino acid esters	CANOPUS
724.609_21.6	$C_{39}H_{77}N_7O_5$	$[M+H]^+$	N-acyl amines	CANOPUS
587.293_5.1	$C_{26}H_{42}N_4O_{11}$	$[M+H]^+$	TAFC precursor	FBMN
760.512_22.7	$C_{40}H_{74}NO_{10}P$	$[M+H]^+$	2-amino-3-({[3-(hexadecanoyloxy)-2-[octadeca-9,12-dienoyloxy]propoxy](hydroxy)phosphoryl}oxy)propanoic acid	GNPS Library
454.352_13.6	$C_{26}H_{44}O_5$	$[M+NH_4]^+$	Fatty acid esters	CANOPUS
266.639_12.7	-	-	-	-
268.264_14.7	$C_{17}H_{33}NO$	$[M+H]^+$	N-acyl amines	CANOPUS
750.624_22.6	$C_{12}H_{10}N_2O$	$[M+H]^+$	4-aminoquinolines	CANOPUS
330.275_12.9	$C_{17}H_{35}N_3O_3$	$[M+H]^+$	Fragin+4(CH_2)	FBMN
760.512_21.3	$C_{40}H_{74}NO_{10}P$	$[M+H]^+$	2-amino-3-({[3-(hexadecanoyloxy)-2-[octadeca-9,12-dienoyloxy]propoxy](hydroxy)phosphoryl}oxy)propanoic acid	GNPS Library
469.682_5.9	$C_{17}H_{29}N_5O_{10}$	$[M–H_2O+Na]^+$	TAFC analog	MS2LDA
461.298_12.0	$C_{24}H_{42}N_2O_5$	$[M+Na]^+$	-	SIRIUS
360.217_10.3	$C_{21}H_{29}NO_4$	$[M+H]^+$	Benzene and substituted derivatives	CANOPUS
692.510_22.6	$C_{40}H_{69}NO_8$	$[M+H]^+$	N-acyl-alpha amino acids	CANOPUS
762.624_19.0	$C_{46}H_{85}NO_8$	$[M–H_2O+H]^+$	Glycerolipids	CANOPUS
279.165_2.7	$C_{10}H_{22}N_4O_5$	$[M+H]^+$	Alpha amino acids	CANOPUS
542.454_19.9	$C_{30}H_{63}O_6$	$[M+Na]^+$	-	SIRIUS
734.593_22.0	$C_{44}H_{79}NO_7$	$[M+H]^+$	Triacylglycerols	CANOPUS
764.639_19.5	$C_{46}H_{85}NO_7$	$[M+H]^+$	Fatty Acyls	CANOPUS
736.609_15.4	$C_{44}H_{81}NO_7$	$[M+H]^+$	Triacylglycerols	CANOPUS
279.165_1.4	$C_{10}H_{22}N_4O_5$	$[M+H]^+$	Alpha amino acids and derivatives	CANOPUS
507.454_19.4	$C_{31}H_{58}N_2O_3$	$[M+H]^+$	Lipids and lipid-like molecules	CANOPUS
279.165_2.1	$C_{10}H_{22}N_4O_5$	$[M+H]^+$	Alpha amino acids and derivatives	CANOPUS
393.314_15.8	$C_{28}H_{42}O_2$	$[M–H_2O+H]^+$	Triterpenoids	CANOPUS
525.498_19.5	$C_{32}H_{64}N_2O_3$	$[M+H]^+$	Trialkylamines	CANOPUS
747.584_21.4	$C_{47}H_{80}O_5$	$[M+Na]^+$	1,2-diacylglycerols	CANOPUS
490.425_18.3	$C_{31}H_{55}NO_3$	$[M+H]^+$	N-acyl amines	CANOPUS
391.299_17.0	$C_{23}H_{44}O_2$	$[M+K]^+$	-	SIRIUS
493.328_16.5	$C_{30}H_{46}O_4$	$[M+Na]^+$	-	SIRIUS

652.479_22.5	$C_{33}H_{61}N_7O_6$	$[M+H]^+$	Oligopeptides	CANOPUS
391.299_17.4	$C_{28}H_{38}O$	$[M+H]^+$	Diterpenoids	CANOPUS

14 metabolite features detected exclusively in co-culture samples in the presence of trimethoprim

*m/z*_RT	Formula	Adduct	Annotation / compound class	Source
360.249_9.5	$C_{17}H_{33}N_3O_5$	$[M+H]^+$	Fragin+4(CH_2)+2(OH)–2H	FBMN
330.251_9.5	$C_{15}H_{31}N_5O_3$	$[M+H]^+$	Fragin+4(CH_2)+2(OH)–2H–NO	FBMN
332.254_8.1	$C_{16}H_{33}N_3O_4$	$[M+H]^+$	Carboxylic acids and derivatives	CANOPUS
382.231_9.5	$C_{17}H_{33}N_3O_5$	$[M+Na]^+$	Fragin+4(CH_2)+2(OH)–2H	FBMN
509.410_19.9	$C_{33}H_{52}N_2O_2$	$[M+H]^+$	Ergosterol related	FBMN
510.430_19.4	$C_{34}H_{55}NO_2$	$[M+H]^+$	Ergosterol related	FBMN
510.430_19.0	$C_{34}H_{55}NO_2$	$[M+H]^+$	Ergosterol related	FBMN
514.425_18.3	$C_{33}H_{55}NO_3$	$[M+H]^+$	Long-chain ceramides	CANOPUS
270.104_2.0	$C_9H_{17}N_3O_5$	$[M+Na]^+$	-	SIRIUS
382.231_8.7	$C_{17}H_{33}N_3O_5$	$[M+Na]^+$	Peptides	CANOPUS
332.235_10.4	-	-	-	-
454.171_6.4	-	-	TAFC analog	FBMN
520.240_6.3	-	-	TAFC analog	FBMN
287.229_11.1	$C_{15}H_{30}N_2O_3$	$[M+H]^+$	Fatty Acyls	CANOPUS

16 metabolite features detected exclusively in co-culture samples in the absence of trimethoprim

*m/z*_RT	Formula	Adduct	Annotation / compound class	Source
443.215_6.4	-	-	-	-
382.331_17.4	$C_{23}H_{43}NO_3$	$[M+H]^+$	N-acyl-alpha amino acids	CANOPUS
406.292_19.8	$C_{22}H_{41}NO_4$	$[M+Na]^+$	-	SIRIUS
426.321_12.8	$C_{24}H_{40}O_5$	$[M+NH_4]^+$	Diterpenoids	CANOPUS
448.304_12.8	$C_{24}H_{43}NO_5$	$[M+Na]^+$	-	SIRIUS
476.334_13.6	$C_{23}H_{49}O_8$	$[M+Na]^+$	-	SIRIUS
399.248_9.1	$C_{20}H_{34}N_2O_6$	$[M+H]^+$	N-acyl-alpha amino acids and derivatives	CANOPUS
364.284_14.2	$C_{22}H_{37}NO_3$	$[M+H]^+$	Alpha amino acid esters	CANOPUS
488.407_18.9	$C_{29}H_{55}NO_3$	$[M+Na]^+$	N-acyl amines	CANOPUS
360.180_10.1	$C_{20}H_{25}NO_5$	$[M+H]^+$	Benzenoids	CANOPUS
348.180_8.6	$C_{19}H_{25}NO_5$	$[M+H]^+$	Pyrrolidones	CANOPUS
335.196_6.0	$C_{18}H_{26}N_2O_4$	$[M+H]^+$	Amino acids and derivatives	CANOPUS
496.508_21.5	$C_{32}H_{65}NO_2$	$[M+H]^+$	N-acyl amines	CANOPUS
530.363_18.4	$C_{35}H_{47}NO_3$	$[M+H]^+$	1-hydroxy-2-unsubstituted benzenoids	CANOPUS
391.222_6.6	$C_{24}H_{32}O_3$	$[M+Na]^+$	Steroids and steroid derivatives	CANOPUS
752.605_21.4	$C_{40}H_{77}N_7O_6$	$[M+H]^+$	Ceramides	CANOPUS

Supplementary Table S4. List of known fungal metabolites detected and annotated in this study.

**Compound name**	**Adduct**	**Theoretical *m/z***	**Experimental *m/z***	**ppm error**
Fumigaclavine B	$[M+H]^+$	257.165	257.165	0.8
Fumigaclavine C	$[M+H]^+$	367.238	367.238	0.8
Isofumigaclavine A	$[M+H]^+$	299.175	299.175	1.3
Verruculogen	$[M–H_2O+H]^+$	494.229	494.228	0.4
Fumitremorgin C	$[M+H]^+$	380.197	380.196	1.6
Demethoxyfumitremorgin C	$[M+H]^+$	350.186	350.186	0.3
Tryprostatin A	$[M+H]^+$	382.213	382.212	1.3
Tryprostatin B	$[M+H]^+$	352.202	352.202	0.0
Fumiquinazoline A	$[M+H]^+$	446.182	446.182	0.2
Fumiquinazoline C	$[M+H]^+$	444.167	444.167	0.0
Fumagillin	$[M+H]^+$	459.238	459.238	0.4
Pyripyropene A	$[M+H]^+$	584.249	584.249	0.0
Pseurotin A	$[M+H]^+$	432.165	432.165	0.2
Asperflagin	$[M+H]^+$	489.358	489.357	0.6
Fumihopaside A	$[M+H]^+$	653.426	653.426	0.0
Endocrocin	$[M+H]^+$	315.050	315.050	0.0
Terezine-D	$[M+H]^+$	326.186	326.187	1.5

Supplementary Table S5. Biosynthetic gene clusters present in *Burkholderia cenocepacia* K56-2 and *Aspergillus fumigatus* A1160+ analyzed in this study.

Strain	Cluster	Category of Predicted Cluster	Most Similar Known Cluster	Similarity	Genome Size (Mb)
B. cenocepacia K56-2					7.81
	1	T1PKS			
	2	Terpene			
	3	Homoserine lactone			
	4	Terpene			
	5	NRPS	Pyochelin	100%	
	6	Terpene			
	7	Phosphonate	Phosphinothricin	6%	
	8	Homoserine lactone	Colicin V	1%	
	9	Betalactone, NRPS	Fragin	100%	
	10	Bacteriocin			
	11	Terpene			
	12	Arylpolyene	Polyhydroxyalkanoate	50%	
	13	Terpene			
	14	NRPS	Ornibactin	100%	
A. fumigatus A1160+					29.3
Chromosome 1	1	T1PKS	Pypripyropene A	66%	
	2	NRPS	Nidulanin A	75%	
	3	Terpene			
	4	Betalactone			
	5	NRPS			
Chromosome 2	1	Indole	Fumigaclavine C	36%	
	2	T1PKS	YWA1	100%	
Chromosome 3	1	NRPS			
	2	T1PKS			
	3	Isocyanide-NRP			

	4	NRPS, indole	Hexadehydroastechrome /terezine-D/astechrome	87%
	5	NRPS		
	6	T1PKS, NPRS		
Chromosome 4	1	NRPS-like		
	2	Fungal-RiPP-like		
	3	Terpene	Fumihopaside A/compound 3/21-β-H-hopane-3beta, 22-diol	75%
	4	T1PKS	Trypacidin	71%
Chromosome 5	1	Isocyanide		
	2	T1PKS	Endocrocin	55%
Chromosome 6	1	NRPS		
	2	NRPS-like	Choline	100%
	3	Terpene	Clavaric acid	100%
	4	Isocyanide	BU-4704/xanthocillin-X monomethylether/melanocin E/melanocin F/fumiformamide	71%
Chromosome 7	1	T1PKS		
	2	NRPS	Fumiquinazoline A/fumiquinazoline C/fumiquinazoline D	100%
	3	NRPS, NRPS-like	Gliovirin	75%
	4	NRPS-like		
	5	NRPS-like	Imizoquin A/imizoquin B/imizoquin C/imizoquin D/TMC-2A/TMC-2B	20%
	6	Fungal-RiPP-like		
Chromosome 8	1	T1PKS, indole	Hancockinone A	66%
	2	Terpene		
	3	Terpene	Squalestatin S1	60%
Chromosome 9	1	NPRS, indole, T1PKS	Verruculogen/fumitremorgin B/fumitremorgin c/tryprostatin A/tryprostatin	66%

		B/demethoxyfumitremorgin C/brevianamide F	
2	NRPS-like		
3	T1PKS	Sartorypyrone	66%
4	Fungal-RiPP-like	Shimalactone A/Shimalactone B	42%

References

(1) Dührkop, K.; Nothias, L.-F.; Fleischauer, M.; Reher, R.; Ludwig, M.; Hoffmann, M. A.; Petras, D.; Gerwick, W. H.; Rousu, J.; Dorrestein, P. C.; et al. Systematic classification of unknown metabolites using high-resolution fragmentation mass spectra. *Nature Biotechnology* **2021**, *39* (4), 462-471. DOI: 10.1038/s41587-020-0740-8.

(2) Wang, M.; Carver, J. J.; Phelan, V. V.; Sanchez, L. M.; Garg, N.; Peng, Y.; Nguyen, D. D.; Watrous, J.; Kapono, C. A.; Luzzatto-Knaan, T.; et al. Sharing and community curation of mass spectrometry data with Global Natural Products Social Molecular Networking. *Nature Biotechnology* **2016**, *34* (8), 828-837. DOI: 10.1038/nbt.3597.

(3) Dührkop, K.; Fleischauer, M.; Ludwig, M.; Aksenov, A. A.; Melnik, A. V.; Meusel, M.; Dorrestein, P. C.; Rousu, J.; Böcker, S. SIRIUS 4: a rapid tool for turning tandem mass spectra into metabolite structure information. *Nature Methods* **2019**, *16* (4), 299-302. DOI: 10.1038/s41592-019-0344-8.

(4) Jenul, C.; Sieber, S.; Daeppen, C.; Mathew, A.; Lardi, M.; Pessi, G.; Hoepfner, D.; Neuburger, M.; Linden, A.; Gademann, K.; et al. Biosynthesis of fragin is controlled by a novel quorum sensing signal. *Nature Communications* **2018**, *9* (1), 1297. DOI: 10.1038/s41467-018-03690-2.

(5) Bittremieux, W.; Chen, C.; Dorrestein, P. C.; Schymanski, E. L.; Schulze, T.; Neumann, S.; Meier, R.; Rogers, S.; Wang, M. Universal MS/MS Visualization and Retrieval with the Metabolomics Spectrum Resolver Web Service. *bioRxiv* **2020**, 2020.2005.2009.086066. DOI: 10.1101/2020.05.09.086066.

(6) Bastos, R. W.; Akiyama, D.; Reis, T. F. d.; Colabardini, A. C.; Luperini, R. S.; Castro, P. A. d.; Baldini, R. L.; Fill, T.; Goldman, G. H. Secondary Metabolites Produced during *Aspergillus fumigatus* and *Pseudomonas aeruginosa* Biofilm Formation. *mBio* **2022**, *13* (4), e01850-01822. DOI: 10.1128/mbio.01850-22.

(7) Ostertag, J. Nachweis und Vorkommen von *Aspergillus fumigatus*-Toxinen in Gras- und Maissilagen Ph.D. Thesis, Technische Universität München, 2010. https://mediatum.ub.tum.de/842068 (accessed 2023-06-20).

(8) Varga, J. J.; Losada, L.; Zelazny, A. M.; Kim, M.; McCorrison, J.; Brinkac, L.; Sampaio, E. P.; Greenberg, D. E.; Singh, I.; Heiner, C.; et al. Draft Genome Sequences of *Burkholderia cenocepacia* ET12 Lineage Strains K56-2 and BC7. *Genome Announcements* **2013**, *1* (5), 10.1128/genomea.00841-00813. DOI: 10.1128/genomea.00841-13.

(9) Bertuzzi, M.; van Rhijn, N.; Krappmann, S.; Bowyer, P.; Bromley, M. J.; Bignell, E. M. On the lineage of *Aspergillus fumigatus* isolates in common laboratory use. *Medical Mycology* **2020**, *59* (1), 7-13. DOI: 10.1093/mmy/myaa075 (acccessed 8/8/2024).

(10) Ferreira, M. E. d. S.; Kress, M. R. V. Z.; Savoldi, M.; Goldman, M. H. S.; Härtl, A.; Heinekamp, T.; Brakhage, A. A.; Goldman, G. H. The *akuB*^{KU80} Mutant Deficient for Nonhomologous End Joining Is a Powerful Tool for Analyzing Pathogenicity in *Aspergillus fumigatus*. *Eukaryotic Cell* **2006**, *5* (1), 207-211. DOI: 10.1128/ec.5.1.207-211.2006.

(11) Tsai, H.-F.; Wheeler, M. H.; Chang, Y. C.; Kwon-Chung, K. J. A Developmentally Regulated Gene Cluster Involved in Conidial Pigment Biosynthesis in *Aspergillus fumigatus*. *Journal of Bacteriology* **1999**, *181* (20), 6469-6477. DOI: 10.1128/jb.181.20.6469-6477.1999.

(12) Tsai, H.-F.; Washburn, R. G.; Chang, Y. C.; Kwon-Chung, K. J. *Aspergillus fumigatus arp1* modulates conidial pigmentation and complement deposition. *Molecular Microbiology* **1997**, *26* (1), 175-183. DOI: 10.1046/j.1365-2958.1997.5681921.x.

(13) Hamad, M. A.; Zajdowicz, S. L.; Holmes, R. K.; Voskuil, M. I. An allelic exchange system for compliant genetic manipulation of the select agents *Burkholderia pseudomallei* and *Burkholderia mallei*. *Gene* **2009**, *430* (1), 123-131. DOI: 10.1016/j.gene.2008.10.011.
